# Metabolic networks in the tumor microenvironment: roles of amino acid and lipid metabolism pathways in cancer progression and therapy

**DOI:** 10.1038/s12276-026-01697-0

**Published:** 2026-04-30

**Authors:** Yulseung Sung, Dae Kyoung Kim, Jun Se Kim, Seong-Jang Kim, Jae Ho Kim, Jung Min Han

**Affiliations:** 1 Yonsei Institute of Pharmaceutical Sciences, College of Pharmacy, Yonsei University, Incheon, South Korea; 2 Department of Physiology, Pusan National University School of Medicine, Yangsan, South Korea; 3 Department of Integrated OMICS for Biomedical Science, Yonsei University, Seoul, South Korea; 4 POSTECH Biotech Center, Pohang University of Science and Technology, Pohang, South Korea; 5 Department of Integrated Biotechnology, Yonsei University, Incheon, South Korea

**Keywords:** Biochemistry, Cancer metabolism, Cancer microenvironment

## Abstract

Metabolic rewiring, a defining hallmark of cancer, sustains cell proliferation and biosynthesis while coordinating adaptive interactions within the tumor microenvironment (TME). Recent advances reveal that metabolism in the TME-comprising stromal, immune and endothelial components forms a complex metabolic network in which intercellular competition, cooperation and plasticity profoundly influence tumor progression and therapeutic responses. Here we integrate emerging evidence on the organizational principles of amino acid and lipid metabolism within the TME, emphasizing how nutrient fluxes shape immune evasion, therapeutic resistance and metabolic symbiosis. We highlight key mechanisms through which cancer and nonmalignant cells engage in reciprocal nutrient manipulation, focusing on glutamine, arginine, tryptophan, branched-chain amino acids and lipids. The dual roles of these metabolites in immune regulation and tumor growth reveal the limitations of traditional single-pathway targeting and advocate for a network-centric therapeutic approach. We further discuss how metabolite-derived signaling and epigenetic regulation reinforce cell state transitions and immune suppression. Current and emerging therapeutic strategies, including multitarget combinations and immune–metabolic synergies, are evaluated alongside translational challenges. Finally, we underscore the need for spatial metabolomics, liquid biopsy platforms and artificial intelligence-driven modeling to map nutrient competition and cooperative exchange within the TME, offering new opportunities for precision metabolic interventions.

## Metabolic networks shape cancer progression and therapy response

Cancer cells need a lot of energy and nutrients to grow, and they often change their metabolism to meet these needs. This review explores how cancer cells and their surrounding environment, called the tumor microenvironment, interact metabolically. The tumor microenvironment includes various cells such as immune cells and fibroblasts, which can influence cancer growth and treatment resistance. Numerous researchers found that cancer cells compete with immune cells for nutrients such as amino acids and lipids. This competition can weaken the immune system’s ability to fight cancer. For example, cancer cells consume more glutamine, an amino acid crucial for both tumor growth and immune cell function. Similarly, lipids are used by cancer cells for energy and building cell membranes, which helps them survive under stress. These studies suggests that targeting these metabolic pathways could improve cancer treatment.

This summary was initially drafted using artificial intelligence, then revised and fact-checked by the author.

## Introduction

The hallmarks of cancer encompass diverse biological capabilities acquired during tumorigenesis, among which, metabolic rewiring has emerged as a fundamental and unifying feature^1,2^. Early studies revealed that the heightened metabolic demand of proliferating cancer cells underlies the efficacy of classical cytotoxic agents such as cisplatin and 5-fluorouracil^3^, reflecting the centrality of nucleotide and biosynthetic metabolism in tumor growth. Otto Warburg’s discovery of aerobic glycolysis reframed metabolism as an active driver, rather than a passive consequence, of oncogenesis^4^. Beyond these well-characterized intrinsic alterations, an intricate metabolic network within the tumor microenvironment (TME) has gained recognition as a key determinant of malignancy and therapeutic escape. The stromal, immune and endothelial compartments not only adapt their own metabolic programs but also engage in nutrient competition, symbiosis and reciprocal regulation that collectively reshape tumor behavior^5–7^.

The classical Warburg effect provided the conceptual foundation linking glycolysis to tumor biology, yet subsequent work revealed that cancer cells flexibly engage glutaminolysis, lipid biosynthesis and other pathways to sustain biosynthesis and redox homeostasis. These changes, however, cannot be understood in isolation^8^. Tumors exist within a heterogeneous ecosystem composed of fibroblasts, immune cells, endothelial cells and extracellular matrix (ECM)^5^. Through nutrient competition, metabolite exchange and paracrine signaling, these compartments cooperate and compete to support tumor growth, immune evasion and therapeutic resistance^6^. Within this complex environment, amino acids and lipid metabolism have emerged as particularly influential axes. Amino acids govern signaling and epigenetic regulation through nutrient competition and metabolite-derived modifications, whereas lipids sustain energy storage, membrane synthesis and ferroptosis sensitivity. Together, they define the metabolic organization of the TME and represent central therapeutic targets.

Despite progress in targeting metabolic pathways, clinical translation has been limited because current strategies seldom account for the bidirectional interplay between cancer and immune cells^3,9^. Agents that modulate nutrient flux often exert dual and sometimes opposing effects-for instance, glutamine antagonists inhibit tumor metabolism yet enhance antitumor immunity, whereas arginine depletion suppresses tumor growth but impairs T cell activation. These dualities underscore the need for a network-centric therapeutic perspective that integrates metabolic and immunologic dimensions.

In this Review, we synthesize current knowledge on the organization of metabolic networks within the TME, focusing on amino acid and lipid metabolism as central axes of nutrient competition, intercellular symbiosis and therapeutic resistance. We discuss how stromal, immune and endothelial compartments collectively shape metabolic circuits that influence tumor progression and treatment outcomes. Finally, we highlight emerging strategies and translational challenges for developing network-based interventions that disrupt metabolic cooperation to improve patient responses.

## Principles of metabolic organization in the TME

### Metabolic plasticity beyond cancer cells

**Fig. 1:**
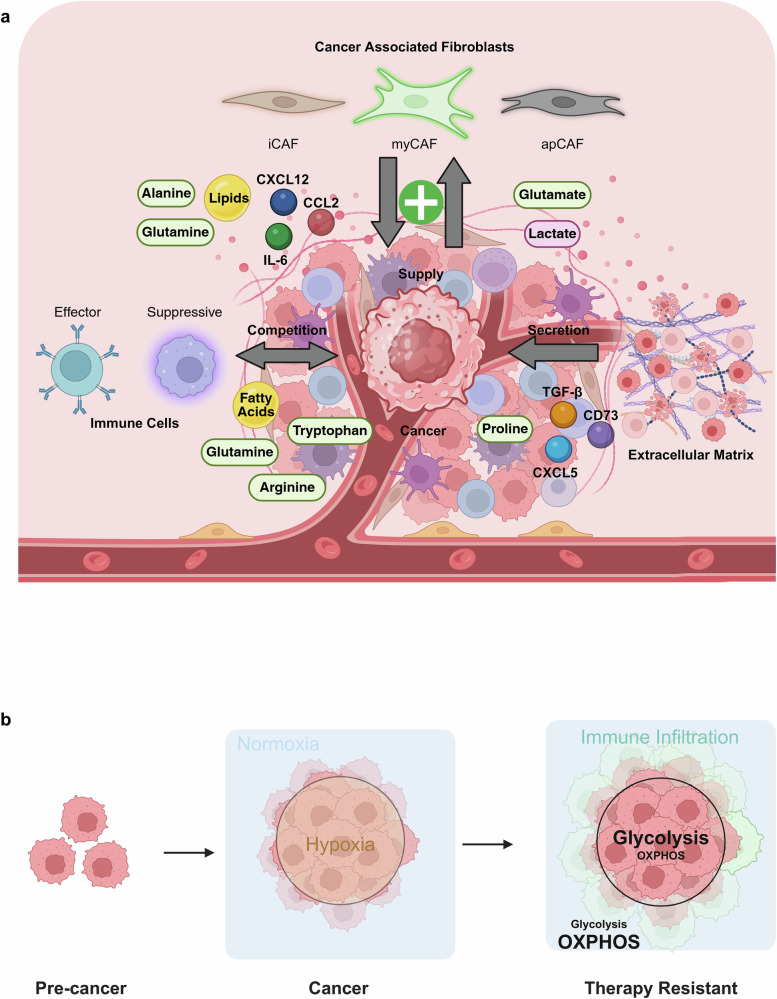
Metabolic plasticity and spatiotemporal heterogeneity in the TME. **a** Distinct subsets of CAFs, iCAFs, myCAFs and apCAFs are stimulated by the metabolites of cancer cells and supply different metabolites and secrete cytokines that drive tumor progression. Cancer cells stimulate CAFs with glutamate or lactate, which results in the supply of alanine, glutamine or lipids. In addition, inflammatory cytokines such as CXCL12, IL-6 and CCL2 are secreted to fuel cancer cells. Cancer cells also interact with the ECM, which secretes immunosuppressive factors, such as TGF-β, CD73, CXCL5 and proline. Moreover, the metabolic competition with immune cells (T cells, B cells and macrophages) for critical nutrients such as glutamine, arginine, tryptophan and fatty acids further reshapes the immune landscape of the TME. **b** The spatial and metabolic remodeling of tumors occurs during progression. In the precancer stage, the tissue starts to form a distinct core-and-periphery architecture. As tumors become malignant, a hypoxic core and oxygenated peripheral regions develop. The hypoxic core is supported mainly by increased glycolysis, and the oxygenated peripheral region is supported mainly through OXPHOS, indicating spatial heterogeneity. In advanced therapy-resistant tumors, metabolic heterogeneity arises, with the hypoxic core reintegrating OXPHOS and glycolysis to resist therapeutic interventions.

The TME is a complex metabolic network composed of cancer cells, stromal and immune cells, vasculature and ECM components^5^. Although cancer cell-intrinsic metabolic rewiring has been extensively characterized, it is increasingly evident that the metabolic behaviors of nonmalignant cells critically influence tumor progression, metastasis and therapeutic response. Stromal cells, particularly cancer-associated fibroblasts (CAFs), and diverse immune cell populations engage in dynamic metabolic crosstalk with cancer cells through the exchange of metabolites, cytokines and growth factors. These interactions generate interdependent metabolic circuits that can either promote tumor growth or potentiate antitumor immunity depending on spatial and temporal context (Fig. 1a).

#### CAFs

CAFs are metabolically heterogeneous and functionally diverse, with subtypes exhibiting distinct phenotypes. Most arise from tissue-resident fibroblasts, but adipocytes, pericytes, endothelial cells and mesenchymal cells also contribute to their activation. Inflammatory CAFs (iCAFs) secrete cytokines such as IL-6, CXCL12 and CCL2 that skew myeloid and T cell populations toward immunosuppressive phenotypes^10^. iCAFs maintain high glycolytic and fatty acid-consuming activity to sustain survival in hypoxia, producing lactate and other metabolites that modulate immune cell function^11^. Myofibroblast-like CAFs (myCAFs) remodel the ECM, increase stromal stiffness and restrict immune infiltration, thereby fostering M2 macrophage polarization and regulatory T cell (T_reg_) expansion^12,13^. These CAFs exhibit active cholesterol metabolism and robust oxidative phosphorylation (OXPHOS) to fuel ECM synthesis. Antigen-presenting CAFs (apCAFs), which express MHC class II molecules but lack costimulatory signals, promote T_reg_ differentiation and immune tolerance^14^. Collectively, CAFs contribute to the nutrient economy of tumors by supplying serine, glycine and proline and by generating aspartate and asparagine during glutamine scarcity, thereby sustaining both stromal and tumor compartments^5^.

#### Immune cells

Immune cell metabolism within the TME is highly context dependent and dynamically linked to activation state. Effector cells—including activated T cells, macrophages, dendritic cells (DCs), neutrophils and B cells—rely on glycolysis to support proliferation and cytokine production^15^. By contrast, regulatory or suppressive immune subsets such as T_reg_ cells, myeloid-derived suppressor cells (MDSCs) and M2 macrophages depend on OXPHOS and fatty acid oxidation (FAO) for maintenance^16,17^. The disruption of redox and mitochondrial homeostasis induces immune exhaustion and limits antitumor function.

Competition for nutrients such as glutamine, arginine and tryptophan defines the immunometabolic landscape of the TME. CAFs and myeloid cells can deplete these amino acids, suppressing cytotoxic T cell activity while promoting T_reg_ expansion^18,19^. CAFs consume glutamine for collagen synthesis but may also release it under stress, whereas immune cells require glutamine for proliferation and effector differentiation^20,21^. Lipid metabolites such as prostaglandin E₂ and lysophosphatidic acid (LPA) further mediate CAF–cancer–immune cell feedback loops that shape immune infiltration and phenotype^22^.

#### ECM and stromal cells

The ECM functions not merely as structural support but as an active regulator of metabolism within the TME. Collagen-rich matrices produced by myCAFs serve both as physical barriers to immune infiltration and as reservoirs of proline and hydroxyproline that influence immune cell metabolism^23^. Matrix stiffness and composition direct macrophage polarization and suppress T cell activation, whereas ECM-bound cytokines and growth factors coordinate metabolic signaling across stromal and immune compartments^24^.

ECM-producing myCAFs and stromal cells also modulate immune checkpoint pathways. Through CD73-mediated adenosine production and the secretion of TGF-β or CXCL5, they inhibit effector T cell metabolism and reduce sensitivity to PD-1/PD-L1 blockade^25^. The synthesis of collagen itself consumes proline and glycine, generating hydroxyproline peptides that feed into the tricarboxylic acid (TCA) cycle. The activation of YAP–TAZ signaling enhances ECM stiffness, glycolysis and glutamine metabolism, establishing a feed-forward loop that reinforces metabolic and structural remodeling.

Collectively, these observations underscore that metabolic plasticity in the TME arises from coordinated interactions among CAFs, immune cells and ECM components. This intercellular metabolic organization provides the foundation for understanding how amino acid and lipid networks govern tumor behavior—a concept explored in the following sections.

#### Metabolic heterogeneity across tumor types and human relevance

A major determinant in generalizing metabolic crosstalk across tumors is the composition of cell types present, including immune subsets and CAFs, as well as the nutrient species available, such as oxygen, amino acids and glucose^26^. These variables are highly dependent on tumor type and patient context, giving rise to distinct metabolic niches and interaction networks. Consequently, tumor metabolism is shaped not only by cell-intrinsic programs but also by tissue-contextual factors and microenvironmental states—for example, vascular supply, hypoxia and stromal density—which collectively determine whether competition or cooperation for specific metabolites predominates. Consistent with this, human tumors frequently exhibit glycolytic reprogramming with tumor type-specific deviations in mitochondrial, lipid and amino acid metabolism that arise from distinct oncogenic drivers and local microenvironmental selection pressures^27,28^.

Multiple studies in tumor patients have revealed how tumor-type context rewires nutrient utilization and remodels stromal and immune relevance. In non-small-cell lung cancer (NSCLC), intraoperative glucose infusion demonstrated that although enhanced glucose metabolism is common, tumors also oxidize lactate with pronounced heterogeneity both within and between tumors, which can be predicted by preoperative perfusion^27^. This highlights how the lung-specific microenvironment generates regionally distinct metabolic states in patients. In prostate cancer, multisubstrate tracing in patient-derived xenografts revealed variable upregulation of glucose, glutamine and FAO across different tumor regions^29^. Spatial multiomics in human bladder cancer further supports organ-specific metabolic rewiring^30^. Choline and glucose metabolism were upregulated, whereas sphingolipid and tryptophan metabolism were suppressed. Such bladder-specific TMEs generated hypoxic regions and promoted immune evasion by stromal exclusion of immune cell infiltration. Even within a single cancer type, metabolic subpopulations can be mapped to resolve heterogeneity, as illustrated in melanoma^28^. Patient-derived melanoma xenograft studies showed that metastatic efficiency was linked to monocarboxylate transporter 1 (MCT1)-dependent lactate metabolism, wherein MCT1 overexpression increased lactate uptake and metastatic potential without altering the phenotype of primary tumor cells.

### Crosstalk and feedback circuits within TME compartments

A defining hallmark of the TME metabolism is the formation of self-reinforcing feedback circuits driven by reciprocal nutrient exchange among cancer cells, stromal populations and infiltrating immune cells^7^. Rather than functioning as isolated metabolic entities, these compartments engage in dynamic intercellular crosstalk that coordinates energy flow, signaling and biosynthesis. Through these interactions, tumors establish metabolic feedback loops that sustain growth, reprogram immune function and foster therapeutic resistance.

Broadly, three archetypal modes of crosstalk define the metabolic organization of the TME:

1. Metabolic competition: cancer and immune cells often compete for key nutrients such as glucose, glutamine and tryptophan. This competition dictates immune fate decisions—for instance, glucose deprivation or tryptophan catabolism suppresses effector T cell activity while expanding regulatory populations.
2. Metabolic cooperation: distinct cell types establish mutualistic exchanges to overcome nutrient scarcity. CAFs secrete lactate, alanine and glutamine that fuel neighboring cancer cells, whereas tumor cells release metabolites such as kynurenine or adenosine that modulate immune and stromal metabolism.
3. Signaling feedback: metabolites themselves act as signaling mediators that reinforce cellular states. Accumulated lactate and succinate stabilize HIF-1α and drive histone modifications, whereas lipid mediators such as prostaglandin E_2_ amplify inflammatory or immunosuppressive feedback circuits.

Together, these interdependent processes form a network-centric metabolic ecosystem, in which nutrient exchange is tightly coupled with signaling and epigenetic control. Understanding these feedback dynamics provides the conceptual basis for the following sections, where we examine how amino acid and lipid metabolic pathways orchestrate tumor–stromal–immune communication within the TME.

#### Bidirectional nutrient manipulation

A recurring feature of these feedback circuits is bidirectional nutrient manipulation, whereby cancer cells not only consume but actively reprogram surrounding stromal and immune cells to supply essential metabolites. Tumor-derived cytokines and signaling molecules can drive fibroblasts and adipocytes to secrete key substrates—such as alanine, glutamine and lipids—that meet the anabolic and energetic demands of proliferating cancer cells^31,32^. These exchanges are inherently reciprocal: metabolites released by tumors, including lactate and glutamate, are taken up by stromal cells as biosynthetic precursors, fueling ECM production and redox maintenance. This reciprocal metabolic coupling closes the loop, establishing a self-sustaining circuit that stabilizes cooperative metabolism within the TME.

#### Metabolic crosstalk with the immune system

A second organizing principle of tumor metabolism is the integration of metabolic pathways with immune regulation. Among these, amino acid metabolism—particularly the arginine and tryptophan pathways—illustrates how stroma–tumor interactions can simultaneously sustain cancer cell proliferation and suppress antitumor immunity. Stromal or myeloid cells deplete essential nutrients such as arginine or tryptophan, thereby starving cytotoxic T cells while producing immunosuppressive metabolites including kynurenine and NO, which reinforce immune tolerance and promote the differentiation of regulatory immune populations^33^. Through these mechanisms, metabolic crosstalk among TME compartments functions as both a growth-promoting and immune-evasive strategy, coupling metabolic adaptation to the establishment of an immunosuppressive niche.

#### Plasticity and resilience

A defining property of these metabolic circuits is their plasticity and resilience, which enable tumors to withstand and adapt to therapeutic stress. For example, stroma-derived cysteine can compensate for therapy-induced nutrient deprivation or oxidative stress, allowing cancer cells to survive conditions that would otherwise be lethal^34^. The redundancy embedded within these intercellular exchanges complicates efforts to target single pathways and underscores the necessity of network-level therapeutic strategies. Although such adaptability poses challenges for effective intervention, it simultaneously reveals new opportunities to exploit context-dependent vulnerabilities in tumor metabolism.

In summary, the principle of crosstalk-driven feedback highlights that TME metabolism operates not as a sum of isolated cellular programs but as an integrated, adaptive web of cooperative and competitive exchanges—one that confers robustness under stress yet remains susceptible to disruption through strategic, systems-based targeting.

### Spatiotemporal heterogeneity and therapeutic escape

Building on the metabolic plasticity of individual cell types, tumor metabolism evolves dynamically across both space and time, influenced by microenvironmental gradients, stromal interactions, clonal selection and therapeutic pressures^5,8^. Hypoxia, nutrient limitation and local signaling cues establish spatially distinct metabolic niches within tumors, whereas temporal factors—such as tumor progression, therapy-induced stress and circadian rhythms—continuously remodel metabolic states. These spatial and temporal variations enable tumors to flexibly alternate between glycolysis and OXPHOS, activate compensatory pathways and select resistant subclones. Understanding these dynamic adaptations is critical for developing therapeutic strategies that anticipate metabolic reprogramming, exploit transient vulnerabilities and ultimately improve treatment durability.

#### Spatial heterogeneity

Tumor glucose metabolism exhibits pronounced spatial heterogeneity, shaped by microenvironmental gradients, stromal interactions and organ-specific adaptations^35^ (Fig. 1b). In hypoxic tumor cores, HIF-1α activation upregulates *GLUT1*, *HK2* and *LDHA*, thereby promoting glycolysis and lactate accumulation^36^. Conversely, oxygen-rich peripheral regions rely on OXPHOS, the TCA cycle and GLS1-dependent glutaminolysis^37^. Stromal components such as CAFs and immune cells contribute to metabolic symbiosis by supplying alternative substrates—glutamine, lactate or ketone bodies—while also competing for shared nutrients.

Metastatic niches further display organ-specific metabolic adaptations: in liver metastases, *MCT1* and *LDHB* are upregulated to facilitate lactate utilization^38^; lung metastases favor OXPHOS and FAO^39^; brain metastases depend on ketone and acetate metabolism via *ACSS2*^40^; and bone metastases remodel the niche through lactate and serine metabolism that promotes osteolysis^41^. Organ-specific metabolic phenotypes in metastasis probably reflect, in part, extrinsic nutrient availability in the target tissue microenvironment; however, recently, quantitative evidence suggests against simple one limiting nutrient leading to one dependency model^42^. Across multiple organs, interstitial fluid nutrient profiles differ markedly from plasma, and nucleotide-related metabolites contributed strongly to between compartment variation, indicating that multiple metabolite classes covary across tissues. In this study, engineered nutrient auxotrophs revealed that single-nutrient abundance alone does not reliably predict tissue-specific metabolic dependencies in metastasis and that metastatic preference is better explained by a combination of multiple nutrient levels together with cell-intrinsic factors^42^.

Recent spatial metabolomics studies of the liver and small intestine illustrate how regional gradients define metabolic cooperation^43^. Deep-learning-assisted analyses revealed that TCA cycle metabolites derived from glutamine and lactate localize to periportal regions of the liver, where high-energy demand also drives AMP accumulation. In the intestine, malate is enriched in villi, whereas citrate is predominant in crypts, indicating that villi engage enhanced glutamine catabolism, whereas crypts utilize lactate oxidation. Moreover, novel matrix-assisted laser desorption–ionization mass spectrometry-based imaging (MALDI-MSI) has enabled tracing of fructose metabolism across hepatic and intestinal compartments, revealing site-specific utilization patterns. Together, these methodological and conceptual advances are uncovering the spatial logic of metabolic cooperation in both physiological and tumor contexts.

#### Temporal heterogeneity

Metabolic states evolve dynamically over the course of tumor progression and treatment. In early stage tumors, glycolysis predominates to sustain rapid proliferation and biosynthesis. As malignancies advance, mitochondrial metabolism can be reactivated through PGC-1α–driven OXPHOS, although glycolytic dependence may persist in specific microenvironments^44^. Therapeutic interventions impose strong selective pressures that reshape metabolic programs: resistance to gefitinib, an EGFR tyrosine kinase inhibitor, is accompanied by the upregulation of glycolytic genes such as *GLUT1*, *HK2* and *LDHA*^45,46^, whereas BRAF inhibitor resistance is linked to enhanced mitochondrial biogenesis and reactivation of OXPHOS^47^.

Circadian regulation further adds a temporal layer to metabolic control. Core clock genes, including *CLOCK*, *BMAL1* and *PER*, interact with oncogenic and hypoxia-responsive pathways to modulate nutrient utilization, redox balance and DNA repair, suggesting opportunities for time-of-day-optimized therapies^48,49^. Dietary interventions also exemplify temporal modulation of metabolism: the fasting-mimicking diet, characterized by cyclic nutrient restriction, enhances immunotherapy efficacy by contracting immunosuppressive myeloid and T_reg_ populations, thereby augmenting cytotoxic immune activity in both preclinical and clinical settings^50^.

Collectively, spatiotemporal heterogeneity in tumor metabolism arises from interconnected metabolic and signaling networks that continuously adapt to environmental stressors. Hypoxia-induced HIF-1α activation promotes glycolysis, lactate accumulation and PDK1-mediated inhibition of pyruvate dehydrogenase, whereas oncometabolites such as succinate and fumarate couple metabolic reprogramming to epigenetic modification^51^. These feedback loops stabilize metabolic phenotypes over time and reinforce malignant progression.

Immunometabolic competition further links metabolism to immune evasion: tumor cells preferentially consume glucose and accumulate lactate and adenosine, thereby suppressing effector T cells, DCs and natural killer (NK) cells while expanding T_reg_ and M2 macrophage populations^52^. Recent findings on histone lysine lactylation reveal how elevated lactate levels directly modulate gene expression in both tumor and stromal compartments^53^. This metabolic–epigenetic coupling underscores how transient metabolic shifts can imprint long-term regulatory states within the tumor ecosystem, providing both mechanistic insight and potential therapeutic entry points.

#### Innovations in tools and translational challenges

Recent methodological advances are transforming how metabolism is interrogated in spatial and temporal dimensions^54^. Spatial metabolomics, powered by high-resolution mass spectrometry imaging, enables the direct mapping of metabolites within intact tissues—preserving regional and cellular context while capturing dynamic metabolic responses^55,56^. Complementary approaches such as stable isotope tracing allow labeled substrates to be followed through metabolic networks, revealing pathway fluxes and, more recently, uncovering previously unrecognized reactions through isotopolog-similarity-based frameworks such as IsoNet^57^. Increasingly, artificial intelligence (AI) and mathematical modeling are being integrated into these analyses to predict carcinogenic metabolites, annotate novel pathways and construct data-driven metabolic networks^58^. Together, these technologies define the spatial organization, dynamic fluxes and predictive architecture of metabolism with unprecedented resolution, positioning the field for transformative biological and translational insights.

Single-cell and spatial technologies have been instrumental in dissecting the multiple layers of tumor metabolic heterogeneity. Single-cell RNA sequencing and spatial transcriptomics uncover cell-specific metabolic phenotypes, whereas metabolic imaging platforms—including fluorodeoxyglucose positron emission tomography, hyperpolarized ^13^C magnetic resonance imaging and matrix-assisted laser desorption–ionization–time of flight mass spectrometry (MALDI-TOF MS)—and fluorescent biosensors such as SoNar and FLII12Pglu enable real-time visualization of metabolic fluxes^59^. Tumor organoids and microfluidic chips provide microenvironment-aware platforms for functional interrogation of metabolic dependencies. Also, AI and machine learning (ML)-based models increasingly aid in predicting optimal therapeutic timing and combination strategies^57^.

Despite these technological advances, substantial translational challenges remain. Imaging approaches must balance spatial resolution with throughput, and current organoid or animal models only partially recapitulate the complexity of human stromal and immune compartments. Circulating metabolite biomarkers often suffer from limited specificity, tissue biopsies may miss intratumoral metabolic heterogeneity and the dense stromal matrix continues to hinder effective drug delivery. Overcoming these barriers will require rational therapeutic sequencing and multimodal combination designs guided by spatiotemporal biomarkers.

Collectively, these insights reveal that tumor glucose metabolism evolves across both space and time, intricately intertwined with immune modulation and epigenetic regulation. Translating this knowledge into therapy demands strategies that integrate cellular context, temporal dynamics and immune state, ideally guided by spatial diagnostics and systems-level modeling. Such an integrated approach provides the conceptual bridge toward the targeted exploration of amino acid and lipid metabolism networks in the TME, which are discussed in the following section.

### Competition and symbiosis in amino acid metabolism

Tumor–immune competition for amino acids: amino acid metabolism represents one of the most critical axes of metabolic interaction between tumor and immune compartments within the TME. Both cancer cells and immune cells rely on overlapping pools of key amino acids—including glutamine, arginine, tryptophan and branched-chain amino acids (BCAAs)—to sustain proliferation, biosynthesis and signaling. Under nutrient-limited conditions, tumor-associated metabolic rewiring frequently deprives immune cells of the substrates required for clonal expansion, effector function and memory formation.

**Fig. 2:**
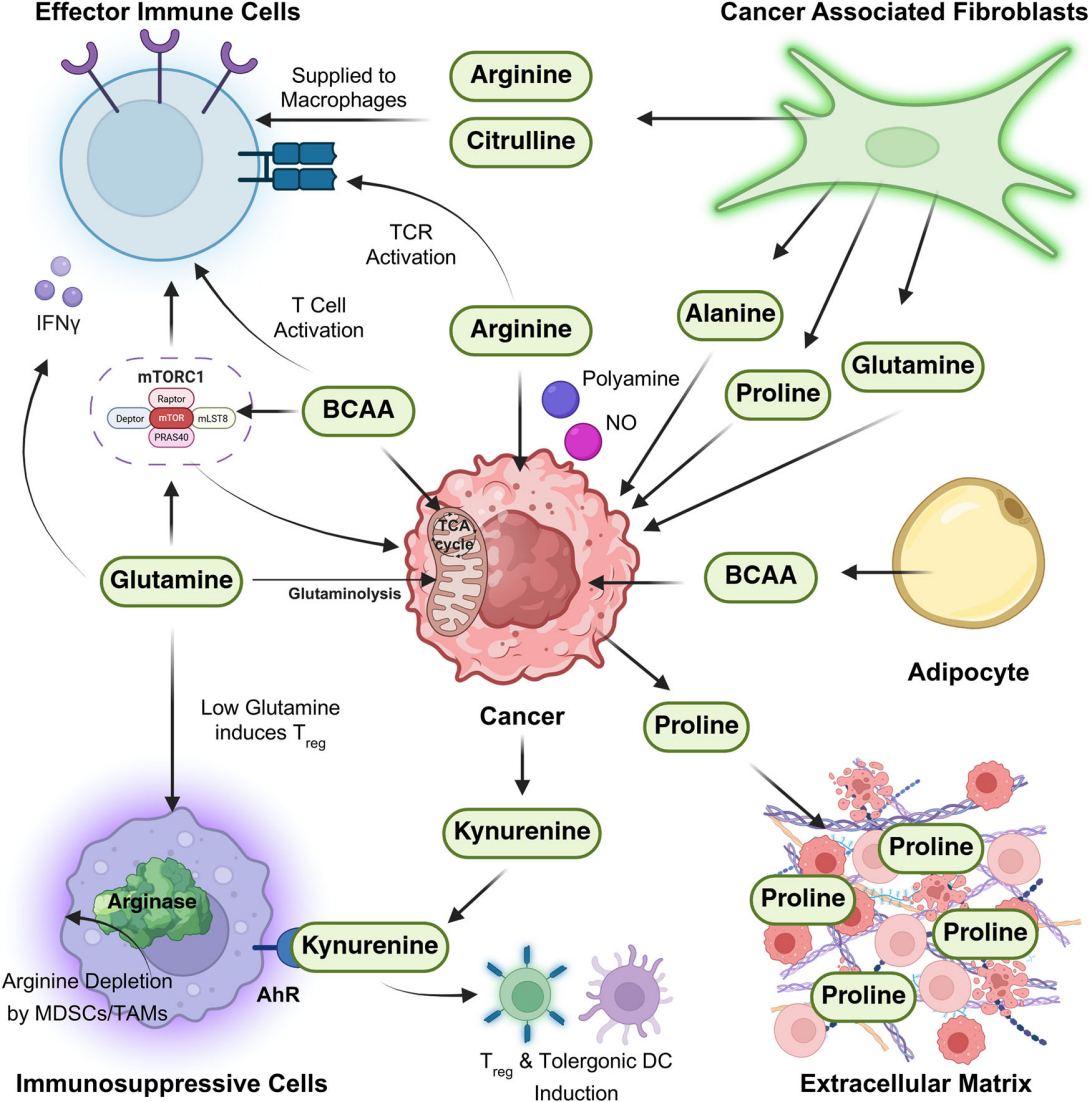
Amino acids in the TME. Cancer cells acquire a diverse pool of amino acids from surrounding stromal and immune cells to support growth, signaling and immune evasion. CAFs provide alanine, glutamine, proline, arginine and citrulline, whereas adipocytes contribute BCAAs. Citrulline and arginine are primarily provided to macrophages, and other metabolites fuel glutaminolysis, the TCA cycle, mTORC1 activation, polyamine biosynthesis and metabolic pathways in tumor cells. Cancer cells secrete proline into the ECM, contributing to remodeling of the microenvironment. Effector immune cells require glutamine, arginine and BCAAs to sustain TCR signaling, proliferation, mTORC1 activation and IFN-γ production. The competition for these nutrients impairs effector function, supporting tumor growth. Glutamine or arginine depletion skews the balance toward immunosuppressive cells. Arginase activity and AHR–kynurenine signaling are exploited by immunosuppressive cells in the TME to promote tolerogenic DC differentiation and T_reg_ induction, which also support tumor growth. Collectively, these reciprocal nutrient exchanges shape a metabolically competitive and immunosuppressive tumor ecosystem.

These shared dependencies generate a state of metabolic competition, wherein cancer cells dominate nutrient uptake through upregulated transporters and adaptive metabolic flexibility. At the same time, the resulting nutrient depletion suppresses antitumor immunity and promotes the emergence of immunosuppressive phenotypes. The following sections highlight major examples of this competition, emphasizing the dual roles of amino acids in both supporting tumor growth and restraining immune activity (Fig. 2).

#### Glutamine

Glutamine represents a central hub of metabolic competition in the TME, serving as both a carbon and nitrogen donor for tumor anabolism and as a critical nutrient for immune effector function^60,61^. Tumor cells frequently exhibit glutamine addiction, importing large quantities of glutamine via transporters such as ASCT2, SNAT2 and LAT1^60,62^. Once internalized, glutamine is transported into mitochondria through SLC1A5_var, where it is catabolized by glutaminases (GLSs) and amidotransferases to generate glutamate, α-ketoglutarate (α-KG), nucleotides, hexosamines and glutathione (GSH)—fueling the TCA cycle, biosynthetic pathways and redox balance^62^.

Oncogenic programs further reinforce this dependency. Myc upregulates *SLC1A5* and *GLS1*, driving glutamine addiction in colorectal, breast and liver cancers. In non-small-cell lung carcinoma and glioblastoma, HIF-1α enhances the expression of glutaminolysis-related genes, whereas in clear cell renal carcinoma, HIF-2α increases the expression of mitochondrial glutamine transporters. Because most nonessential amino acids in cancer cells derive from glutamine, this metabolic rewiring creates a profound dependency that supports malignant proliferation and survival.

In parallel, immune cells also rely heavily on glutamine metabolism. CD8⁺ T cells require glutamine to sustain mTORC1 activation, clonal expansion and effector cytokine production^25,63^. Glutamine-derived GSH synthesis maintains redox balance during T cell activation. However, competition with tumor cells for glutamine results in reduced uptake by T cells, leading to impaired proliferation, cytokine secretion and effector function. Glutamine availability also shapes T helper cell differentiation: supplementation promotes T_h_1 and T_h_17 polarization, whereas deprivation favors T_reg_ development, accompanied by increased FoxP3 expression during TCR engagement^15,21^.

Other immune populations exhibit similar dependency. NK cells require glutamine to sustain Myc expression and meet their high energetic demand^64^. Glutamine depletion leads to reduced NK-cell proliferation, diminished interferon (IFN)-γ secretion and functional exhaustion^64,65^. In macrophages, the ratio of α-KG to succinate dictates polarization—high α-KG promotes an M2 immunosuppressive phenotype, whereas THE inhibition of glutamine synthetase drives M1 activation, reducing immunosuppression and metastatic potential. Thus, glutamine metabolism exerts divergent effects depending on the cellular compartment and metabolic context.

Glutamine depletion overall induces immune evasion when it arises through metabolic competition: highly proliferative tumor cells overexpress glutamine transporters and GLS, outcompeting T cells for extracellular glutamine. Because glutamine is required for mTORC1 activation and IFN-γ production in lymphocytes, its scarcity diminishes T cell metabolic fitness and effector function. Spatially, these effects are most pronounced in tumor cores and perivascular zones, where fluctuating nutrient delivery produces asymmetric immune dysfunction across the tumor mass^66^.

Within CAFs, tumor-derived ammonia, a byproduct of glutaminolysis, induces autophagic flux and mitochondrial stress^10^. This stress response stimulates glutamine secretion, which in turn replenishes tumor cell metabolism, reinforcing a CAF–tumor glutamine cycle that supports proliferation and mitochondrial activity in ovarian and lung adenocarcinoma.

Tumor cells possess remarkable metabolic flexibility, activating alternative glutamine catabolic or anaplerotic pathways when one route is inhibited, whereas immune cells display more rigid metabolic programming^67^. This asymmetry establishes a nutrient hierarchy in the TME, granting tumor cells preferential access to glutamine and forcing immune cells into dysfunctional or exhausted states^68^. In essence, glutamine epitomizes tumor–immune metabolic competition: it is indispensable for both tumor and immune compartments, yet tumor-driven consumption and adaptive metabolic plasticity provide malignant cells with a decisive advantage, undermining the metabolic fitness of antitumor lymphocytes and tipping the balance toward immune evasion and tumor persistence.

#### Arginine

Arginine plays a pivotal role in shaping immunometabolic interactions within the TME, functioning both as a metabolic substrate and a signaling regulator. Although categorized as a semiessential amino acid, arginine becomes conditionally essential in many cancers. Tumor cells frequently downregulate or epigenetically silence argininosuccinate synthase 1 (ASS1), a key enzyme in the urea cycle, thereby shifting their metabolism toward dependence on exogenous arginine uptake^69,70^. Promoter methylation and HIF-1α-mediated transcriptional repression of *ASS1* have been documented in melanoma and hepatocellular carcinoma, establishing a state of arginine auxotrophy. This dependency supports anabolic processes such as protein synthesis, polyamine and nitric oxide (NO) production and contributes to creatine and proline biosynthesis, maintaining energy and redox homeostasis in tumor cells.

The imbalance in arginine availability extends beyond intrinsic tumor demands and profoundly influences immune cell behavior. Cytotoxic CD8⁺ T cells require arginine for activation, proliferation and effector function^71,72^. Within T cells, an active urea cycle generates urea and ornithine, sustaining biosynthetic and proliferative capacity. In the TME, however, MDSCs and tumor-associated macrophages (TAMs) express high levels of arginases (ARG1, ARG2), which deplete extracellular arginine and impair T cell function^73,74^. These enzymes act in concert with inducible NO synthase (iNOS) to convert arginine into reactive nitrogen species, damaging T cell receptor components and suppressing signaling pathways. Nitrosylation of STAT1 and JAK3, for instance, disrupts IFN-γ signaling, weakening antitumor responses.

Conversely, T_reg_ are less sensitive to arginine deprivation, allowing them to persist under nutrient-poor conditions. Pharmacologic arginine inhibitors can therefore exert dual effects—restricting tumor growth while indirectly enhancing effector immune activation. Yet, when arginine is severely depleted, T cell receptor signaling is compromised, leading to CD3ζ downregulation and reduced IFN-γ production^74^. Tumors further exacerbate immune suppression by expressing arginine-metabolizing enzymes such as arginine deiminase (ADI) and arginine decarboxylase (ADC), which generate immunomodulatory metabolites that induce PD-1 and TIM-3 expression, promoting immune exhaustion and evasion. Thus, the immunologic outcome of arginine availability in the TME is highly context dependent.

A form of metabolic symbiosis can also arise between tumors and stromal cells, particularly in ASS1-proficient stromal compartments. In these contexts, stromal cells synthesize and release arginine that can be utilized by adjacent tumor cells, whereas tumors supply stromal partners with ornithine and citrulline^19^. This reciprocal arginine–ornithine cycle fuels polyamine biosynthesis in macrophages, enhancing production of spermine via ornithine decarboxylase (ODC). Spermine, in turn, reinforces immunosuppressive macrophage polarization through p53-mediated DNA demethylation^75^. This spatially organized metabolic exchange exemplifies a typical pattern of symbiosis in which ASS1-positive stromal cells support ASS1-deficient tumor cells.

Collectively, these dynamics establish arginine metabolism as a key node of metabolic competition and cooperation within the TME. Tumor cells exploit arginine for growth and immune evasion, whereas immune cells rely on it for sustaining effector functions. The TME thus represents a battleground of arginine availability, where tumor, stromal and myeloid populations actively modulate nutrient fluxes. Targeting arginine metabolism—either by blocking tumor uptake, modulating arginase activity or disrupting stromal support—offers promising avenues to restore immune competence and sensitize tumors to immunotherapy.

#### Tryptophan

Tryptophan metabolism is a critical determinant of immune regulation within the TME, acting both as a nutrient bottleneck and a source of immunosuppressive metabolites. Tumor cells and myeloid populations—particularly TAMs and DCs—express high levels of tryptophan-catabolizing enzymes such as indoleamine 2,3-dioxygenase (IDO; IDO1 and IDO2) and tryptophan 2,3-dioxygenase (TDO), which deplete extracellular tryptophan and divert it toward kynurenine production^76^. This metabolic diversion limits tryptophan availability for effector T cells, which require it for protein synthesis, proliferation and mTORC1 activation^77^. As a result, T cell proliferation is curtailed, and effector functions are blunted.

Beyond nutrient restriction, the accumulation of tryptophan catabolites, particularly kynurenine, exerts direct immunosuppressive effects through receptor-mediated signaling^78^. Kynurenine is a high-affinity ligand of the aryl hydrocarbon receptor (AHR), a transcription factor that orchestrates the differentiation and function of multiple immune subsets^79^. In T cells, AHR activation favors differentiation toward T_reg_ at the expense of T_h_1 and cytotoxic T cell lineages, reinforcing immune tolerance. Tryptophan depletion, therefore, in the environment, undermines immune competence. Tumor and myeloid cells metabolize tryptophan into kynurenine, which binds and activates the AHR, elevating PD-1 expression on CD8⁺ T cells and inducing apoptosis in DCs^80,81^. These processes amplify immunosuppression through both receptor-mediated signaling and nutrient restriction. In DCs, kynurenine–AHR signaling suppresses antigen presentation and promotes a tolerogenic phenotype, thereby dampening antitumor immunity^82^.

This dual mechanism—tryptophan depletion and kynurenine accumulation—constitutes a potent axis of immune evasion, reinforced by feed-forward loops in which AHR activation upregulates IDO1 expression^80,81^. Elevated kynurenine concentrations correlate with poor prognosis across multiple cancers, including glioblastoma, melanoma and lung carcinoma, underscoring the clinical relevance of this metabolic checkpoint^9^. Spatially, IDO1/TDO expression is often enriched in regions adjacent to vasculature or necrotic cores, suggesting that microenvironmental gradients modulate the degree of immune exclusion^37^.

Recent studies have further expanded this network to include microbiota-derived tryptophan metabolites, such as indole-3-acetate, indole-3-ethanol and indole lactate, which are regulated by gut commensal factors such as RELMβ^82,83^. These metabolites influence T_reg_ differentiation and promote colorectal tumorigenesis, linking systemic metabolism to local immune control^84^. Thus, tryptophan in the TME orchestrates a multifaceted interplay between nutrient competition, metabolic signaling and microbial modulation.

Understanding the role of tryptophan metabolism in immune surveillance is essential for therapeutic innovation. Although IDO1 inhibitors have yielded mixed outcomes in clinical trials—probably owing to redundancy with TDO and compensatory pathways—broader strategies are under exploration. These include dual IDO and TDO blockade, AHR antagonism and modulation of tryptophan transport systems to restore immune responsiveness^3^. Collectively, the tryptophan–kynurenine–AHR axis represents a crucial metabolic bottleneck at the interface of tumor and immune cell interactions, shaping both local and systemic immune landscapes in cancer.

#### BCAAs

In addition to glutamine, arginine and tryptophan, BCAAs—leucine, isoleucine and valine—play pivotal roles in modulating tumor–immune dynamics within the TME. Beyond serving as substrates for protein synthesis, BCAAs function as critical metabolic signals that influence both immune cell activation and tumor proliferation.

BCAA uptake and catabolism are frequently upregulated in cancer cells through the increased expression of the transporter LAT1 (*SLC7A5*) and the enzymes branched-chain aminotransferase 1 and 2 (*BCAT1/2*)^85^. Enhanced BCAA metabolism promotes tumor growth by fueling the TCA cycle via branched-chain α-keto acids and by activating mTORC1 signaling, a central regulator of cell growth and anabolic metabolism^86^. This dependency is particularly pronounced in tumors driven by MYC or KRAS, which coordinately upregulate *LAT1* and *BCAT* expression^87^.

In parallel, immune effector cells within the TME—especially activated CD8⁺ T cells and NK cells—also depend on BCAAs for proliferation and cytokine production^88^. Leucine-dependent mTORC1 activation supports T cell expansion, effector cytokine secretion and memory differentiation^89^. However, competition for BCAAs between tumor and immune compartments can create nutrient-deprived, immunosuppressive conditions, wherein excessive tumor consumption limits BCAA availability to infiltrating T cells, leading to impaired activation and exhaustion.

Moreover, TAMs and T_reg_ exploit BCAA catabolism to sustain their suppressive phenotypes. Both populations exhibit elevated BCAT1 activity, which supports M2 polarization and T_reg_ maintenance while depleting local BCAA pools, thereby indirectly dampening effector immune responses^90^. Notably, the immunologic consequences of BCAA metabolism are context dependent^85^. Under nutrient-replete conditions, BCAAs promote T cell activation; yet, chronic exposure to high levels of BCAAs or their catabolites—such as branched-chain α-keto acids—can induce metabolic stress and T cell exhaustion^91^. This duality underscores the importance of maintaining BCAA homeostasis within the TME and cautions against indiscriminate inhibition of BCAA pathways in therapy.

Overall, BCAA metabolism exemplifies a competitive and context-dependent metabolic axis within the TME, where tumor and immune cells vie for the same essential resources to sustain divergent functional programs. These insights highlight the therapeutic potential of modulating BCAA availability, transporter expression or catabolic enzyme activity to rebalance nutrient competition and enhance antitumor immunity.

### Niche-specific depletion and sharing lead to immune evasion and drug resistance

The metabolic landscape of the TME is shaped not only by nutrient competition but also by strategic nutrient sharing, both of which profoundly influence antitumor immunity and therapeutic response. Within spatially heterogeneous tumor niches—such as the hypoxic core, immune-infiltrated margin, adipose-rich periphery and fibrotic stromal barrier—the availability of amino acids fluctuates dramatically. These gradients create metabolic microdomains that foster selective nutrient depletion or cooperative recycling among cancer, stromal and immune cells. The resulting metabolic stratification generates pockets of immune suppression and drug resistance, illustrating how local nutrient dynamics and intercellular exchange jointly remodel the therapeutic landscape of the TME.

#### Amino acid depletion and drug resistance

The same mechanisms that suppress antitumor immunity through amino acid depletion can also foster resistance to targeted therapies and chemotherapy. Under metabolic stress, tumor cells activate adaptive survival programs that rewire metabolism and attenuate cytotoxic responses. For example, glutamine starvation triggers ATF4-dependent and mTORC1/2-independent stress pathways that enhance antioxidant defenses and induce transient cell-cycle arrest until therapeutic pressure subsides^92^. These adaptations blunt the efficacy of cytotoxic agents that rely on oxidative or mitotic stress, thereby promoting drug resistance.

The depletion of glutamine, cysteine and glutamate—key substrates for GSH synthesis—further contributes to resistance through metabolic compensation. Increased nucleotide biosynthesis drives cisplatin resistance in NSCLC; elevated NADH levels promote gemcitabine resistance in pancreatic ductal adenocarcinoma (PDAC); and enhanced GSH synthesis supports sorafenib resistance in HCC^92^. Similarly, BCAA depletion via increased catabolism promotes energy generation in tamoxifen- and anti-estrogen-resistant breast cancers^86^. BCAT1/2 utilize BCAAs to produce branched-chain acyl-CoA, fueling the TCA cycle and sustaining tumor survival under therapeutic stress. Mechanistically, LLGL2 interacts with LAT1 and YKT6 to augment leucine uptake, resulting in anti-estrogen resistance in breast cancer cells^86^.

In hypoxic niches, tryptophan and arginine depletion synergize with low pH and oxygen tension to induce epithelial–mesenchymal transition (EMT) and resistant phenotypes^93^. EMT transcription factors such as SNAIL and ZEB1 are upregulated in amino acid-restricted regions, reducing drug sensitivity through chromatin remodeling and antiapoptotic signaling^94^. Accordingly, EMT-positive melanoma cells exhibit robust resistance to immune checkpoint blockade and BRAF inhibitors^95^.

Tumor cells can also evade nutrient-targeted therapies through macropinocytosis, a scavenging process that internalizes extracellular proteins and necrotic debris to liberate intracellular amino acids^31^. This compensatory mechanism confers resistance to amino acid-depleting drugs and circumvents transporter-targeted interventions. Within CAF-rich niches, resistance is reinforced by both physical drug diffusion barriers and metabolic quiescence. myCAFs encapsulate tumor cells, buffering against therapeutic agents and maintaining survival under nutrient and drug stress^13^.

Collectively, these findings illustrate that amino acid depletion acts as a double-edged sword—driving immune suppression while simultaneously activating survival pathways that confer metabolic resilience and therapeutic resistance. Understanding these adaptive responses provides a rationale for combination therapies that pair metabolic interventions with targeted or immunotherapeutic agents to overcome treatment failure.

#### Amino acid sharing and drug resistance

Amino acid sharing can also reinforce therapeutic resistance by providing metabolic redundancy that enables tumor cells to withstand nutrient-targeted or cytotoxic therapies. Tumor cells embedded within supportive stromal niches bypass drug-induced amino acid starvation by importing metabolites supplied by neighboring cells. For example, in response to GLS inhibition, CAFs secrete glutamine or alanine, reprogramming central carbon metabolism in tumor cells to restore TCA cycle activity and sustain proliferation despite treatment^96^. In hypoxic or acidic microenvironments where diffusion is restricted, localized recycling intensifies: lactate secreted by glycolytic CAFs fuels oxidative tumor cells, and autophagy-derived amino acids sustain resistant subclones^97^.

Spatially restricted nutrient-sharing phenotypes, in which tumor cells benefit from cooperative metabolic niches, whereas immune cells remain metabolically paralyzed, contribute a distinct form of immune evasion. Extracellular vesicles and exosomes exacerbate this disparity by transferring metabolic enzymes or suppressive cytokines into immune-infiltrated areas, thereby degrading nutrients or remodeling the microenvironment to favor tumor persistence^98^. Several examples highlight the biochemical diversity of amino acid exchange within the TME. Histidine is converted to histamine via histidine decarboxylase (HDC)^99^. Histamine production by mast cells, neutrophils and MDSCs exerts anti-inflammatory and proangiogenic effects, supporting immune escape and tumor progression in glioblastoma and hepatocellular carcinoma^99,100^. Lysine metabolism yields crotonyl-CoA in tumors, and immune cells with elevated lysine catabolism exhibit suppressed type I interferon signaling, generating an immunosuppressed milieu in glioblastoma^101^. Aromatic amino acids such as tyrosine also influence immune regulation: the enzymes 4-hydroxyphenylpyruvate dioxygenase (HPD) and homogentisate 1,2-dioxygenase (HGD) catabolize tyrosine into α-KG, integrating into the TCA cycle. Supplementation with these amino acids correlates with increased PD-L1 expression in glioma cells, linking amino acid excess to checkpoint-mediated immune resistance^102^. Collectively, these examples illustrate how amino acid sharing contributes to immune escape and immunotherapy resistance.

EMT-like states triggered by shared TGF-β and other paracrine factors further enhance resistance by upregulating amino acid transporters and detoxification enzymes, thus protecting tumor cells from both cytotoxic drugs and metabolic inhibitors^103^. Such cooperative remodeling of amino acid metabolism activates therapeutic evasion programs, allowing tumor cells to persist under intercellular nutrient support.

Specific amino acids provide further mechanistic detail. Aspartate, imported via SLC1A2 and SLC1A3, supports nucleotide synthesis and confers resistance to L-asparaginase, a key therapeutic agent in acute myeloid and lymphoblastic leukemia^104^. The upregulation of these transporters maintains nucleotide pools under amino acid-depleting therapy, prompting ongoing clinical trials that combine L-asparaginase with other antimetabolic agents to overcome resistance^104^. Serine contributes to resistance through its role in one-carbon metabolism and folate-mediated nucleotide synthesis, supporting resistance to oxaliplatin in colorectal cancer and doxorubicin in triple-negative breast cancer^105^. Elevated uptake via SLC6A14, shared by immune and epithelial cells^106^, sustains proliferative capacity under chemotherapeutic stress. Histidine metabolism also intersects with methotrexate resistance: the histidine-catabolizing enzyme histidine ammonia-lyase (HAL) consumes folate derivatives, sensitizing tumor cells to methotrexate. Conversely, the loss of HAL or formimidoyltransferase cyclodeaminase (FTCD) preserves tetrahydrofolate (THF) and nucleotide synthesis, conferring methotrexate resistance in lymphoma and leukemia^107^.

Collectively, these findings demonstrate that amino acid sharing and metabolic cooperation establish reservoirs of resistant subpopulations within the TME. By buffering against therapeutic stress and sustaining biosynthetic flux, intercellular exchange networks maintain tumor viability and seed relapse, underscoring the need for combination strategies that disrupt nutrient support alongside targeted or cytotoxic therapies.

### Epigenetics and signaling roles of amino acid metabolism

Amino acids, long regarded merely as precursors for protein synthesis and energy production, have emerged as central regulators of epigenetic and signaling networks within the TME. By donating key functional groups or serving as substrates and cofactors for chromatin-modifying and signaling enzymes, amino acid-derived metabolites link cellular metabolism to transcriptional regulation and epigenetic reprogramming. Through these connections, they modulate tumor plasticity, immune evasion and therapeutic resistance.

This section examines how amino acid metabolism exerts noncanonical regulatory functions—shaping histone and DNA modification states, signaling cascades and cell fate transitions—and highlights their importance in establishing metabolic–epigenetic coupling within the tumor ecosystem.

#### Serine and glycine

Serine and glycine are central components of the one-carbon metabolism network, supplying the one-carbon units required for the generation of *S*-adenosylmethionine (SAM)—the universal methyl donor for DNA and histone methylation. Serine is converted to glycine by serine hydroxymethyltransferase (SHMT), which transfers a one-carbon unit to THF to form 5,10-methylene-THF. This intermediate feeds into the folate and methionine cycles, ultimately producing SAM^105^. The intracellular SAM pool is thus dynamically regulated by serine and glycine availability.

In nutrient-limited tumors, serine deprivation leads to reduced SAM synthesis and global DNA hypomethylation, resulting in epigenetic reprogramming that enhances stemness, plasticity and therapeutic resistance^108^. For example, the elevated expression of PSPH and SHMT1, key enzymes in serine and glycine metabolism, enhances the recruitment of M2 macrophages, immunosuppressive NK cells and memory CD4⁺ T cells^106^. Conversely, serine- and glycine-deprived environments reduce PD-1 expression and disrupt T cell signaling, facilitating immunotherapy resistance.

These methylation dynamics create context-dependent vulnerabilities, and DNA methylation itself has emerged as a therapeutic target in several malignancies, including breast and lung cancers^109^.

#### Glutamine and α-KG

Glutamine metabolism through glutaminolysis generates glutamate and subsequently α-KG, a key cofactor for a broad class of α-KG–dependent dioxygenases^60^. These enzymes include the ten–eleven translocation (TET) family of DNA demethylases and the Jumonji C (JmjC) domain-containing histone demethylases (KDMs), which catalyze the oxidative demethylation of 5-methylcytosine and methylated histone lysines, respectively. Thus, α-KG availability—regulated by glutamine flux, mitochondrial metabolism and redox balance—directly governs chromatin accessibility, gene expression and cellular differentiation states^62,110^.

Conversely, tumor hypoxia and metabolic rewiring often lead to the accumulation of fumarate and succinate, which act as competitive inhibitors of α-KG–dependent dioxygenases. This inhibition stabilizes a hypermethylated, dedifferentiated chromatin state that supports tumor progression and therapeutic resistance^60,111^.

#### Arginine and polyamines

Arginine metabolism contributes to epigenetic regulation through its conversion to polyamines—putrescine, spermidine and spermine—via ODC. These polyamines bind DNA with high affinity, promoting nucleic acid stability, chromatin compaction and transcriptional control^112^. Beyond their structural roles, polyamines also modulate histone acetylation and broader chromatin dynamics. Spermidine, in particular, has been shown to inhibit histone acetyltransferases (HATs) and alter the acetylation levels of key transcriptional regulators^113,114^.

These findings delineate a direct metabolic–epigenetic interface, whereby arginine catabolism and polyamine biosynthesis remodel chromatin topology to reinforce oncogenic transcriptional programs and sustain tumor growth.

#### BCAAs and mTORC1 signaling

BCAAs, particularly leucine, act as potent activators of the mTORC1 pathway, which serves as a key integrator of nutrient status, anabolic signaling and epigenetic control^115^. mTORC1 activation triggers the phosphorylation of downstream effectors such as S6 kinase (S6K) and 4E-binding protein 1 (4EBP1), promoting anabolic gene expression, protein synthesis and histone biosynthesis. In addition, mTORC1 influences the activity and localization of epigenetic enzymes, including HATs and TET DNA demethylases^116^.

Nutrient-driven mTORC1 activation also elevates acetyl-CoA levels, enhancing global histone acetylation and transcriptional accessibility^117^. Through this coupling of amino acid abundance to chromatin modification, BCAA availability dynamically links metabolic inputs with epigenetic and transcriptional outputs, thereby coordinating tumor cell growth, adaptation and resistance within the nutrient-variable microenvironment.

## Heterogeneity of lipid metabolism in cancer and emerging therapeutic targets in the TME

Metabolic reprogramming is a hallmark of cancer and is essential for sustaining cellular growth, proliferation and survival^118^. Although the Warburg effect, characterized by aerobic glycolysis, has long been recognized, recent attention has turned toward lipid metabolism, which plays equally critical roles in cancer pathophysiology^119^. Lipids function not only as structural components of cellular membranes but also as energy reservoirs and secondary signaling molecules that regulate growth and stress adaptation^119,120^.

Dysregulated lipid metabolism enables cancer cells to thrive under nutrient limitation, hypoxia and other metabolic stresses imposed by the TME^120^. Within this ecosystem, lipid metabolism exhibits profound spatiotemporal and cellular heterogeneity, varying across tumor types and among the distinct populations of cancer cells, stromal fibroblasts, adipocytes and immune cells^119,121^. Each compartment engages in unique lipid metabolic programs involving fatty acids, cholesterol and phospholipids, which collectively shape tumor growth and immune modulation^22,122^. Altered lipid utilization by CAFs, adipocytes and immune cells profoundly remodels the metabolic landscape of the TME^121^.

Understanding this complex and dynamic lipid metabolic interplay is crucial for identifying vulnerabilities and developing next-generation diagnostic and therapeutic strategies tailored to the metabolic heterogeneity of cancer.

### Intratumoral lipid metabolism in cancer cells

Cancer cells exhibit markedly elevated de novo fatty acid synthesis and enhanced uptake of exogenous fatty acids from the TME to sustain their high demands for biomass production and energy supply^121^. This metabolic reprogramming supports proliferation, angiogenesis and metastatic dissemination^123^. A central feature of this adaptation is the strategic utilization of acetyl-CoA, a pivotal hub linking glucose and lipid metabolism, to drive lipid synthesis and remodeling^122,124^.

Elevated fatty acid synthase (FASN) and stearoyl-CoA desaturase 1 (SCD1) activity represents a hallmark of many cancer types, enabling continuous lipid production for membrane biogenesis and redox maintenance^125^. These pathways are transcriptionally governed by sterol regulatory element-binding protein 1 (SREBP1), which coordinates lipid anabolism in response to nutrient and growth factor cues^126^. Conversely, FAO—mediated by carnitine palmitoyltransferase 1A (CPT1A) in mitochondria—is upregulated in several cancers, including breast, prostate, colorectal and glioblastoma, to sustain ATP production and redox balance under metabolic stress^127^.

FAO is particularly critical for cancer stem cells (CSCs), where CPT1A-driven oxidation supports self-renewal and chemoresistance^128^. In triple-negative breast cancer, CPT1A cooperates with acyl-CoA synthetase long-chain family member 4 (ACSL4) to promote FAO and lipid droplet (LD) accumulation, which buffer oxidative stress and confer drug tolerance^129^. The balance between fatty acid synthesis and oxidation is tightly controlled by peroxisome proliferator-activated receptor α (PPAR-α), integrating metabolic flux with proliferation and survival signals^130^.

Cholesterol metabolism is another key axis of lipid reprogramming. Cholesterol accumulation correlates with tumor aggressiveness, notably in prostate cancer, and is regulated by the liver X receptor (LXR) and the mevalonate pathway, which together maintain intracellular cholesterol and isoprenoid homeostasis^131–133^. Tumors lacking FASN activity often exhibit compensatory activation of the mevalonate pathway, underscoring the metabolic redundancy that supports lipid anabolism and therapeutic resistance^133^.

Collectively, these intratumoral lipid programs converge on sustaining membrane biogenesis, redox homeostasis and metabolic plasticity, which collectively underpin tumor growth and therapy tolerance. The future integration of flux analyses, single-cell lipidomics and spatial metabolomics will be crucial for identifying context-specific vulnerabilities and refining therapeutic target selection.

### Lipid metabolism in stromal and immune cells

**Fig. 3:**
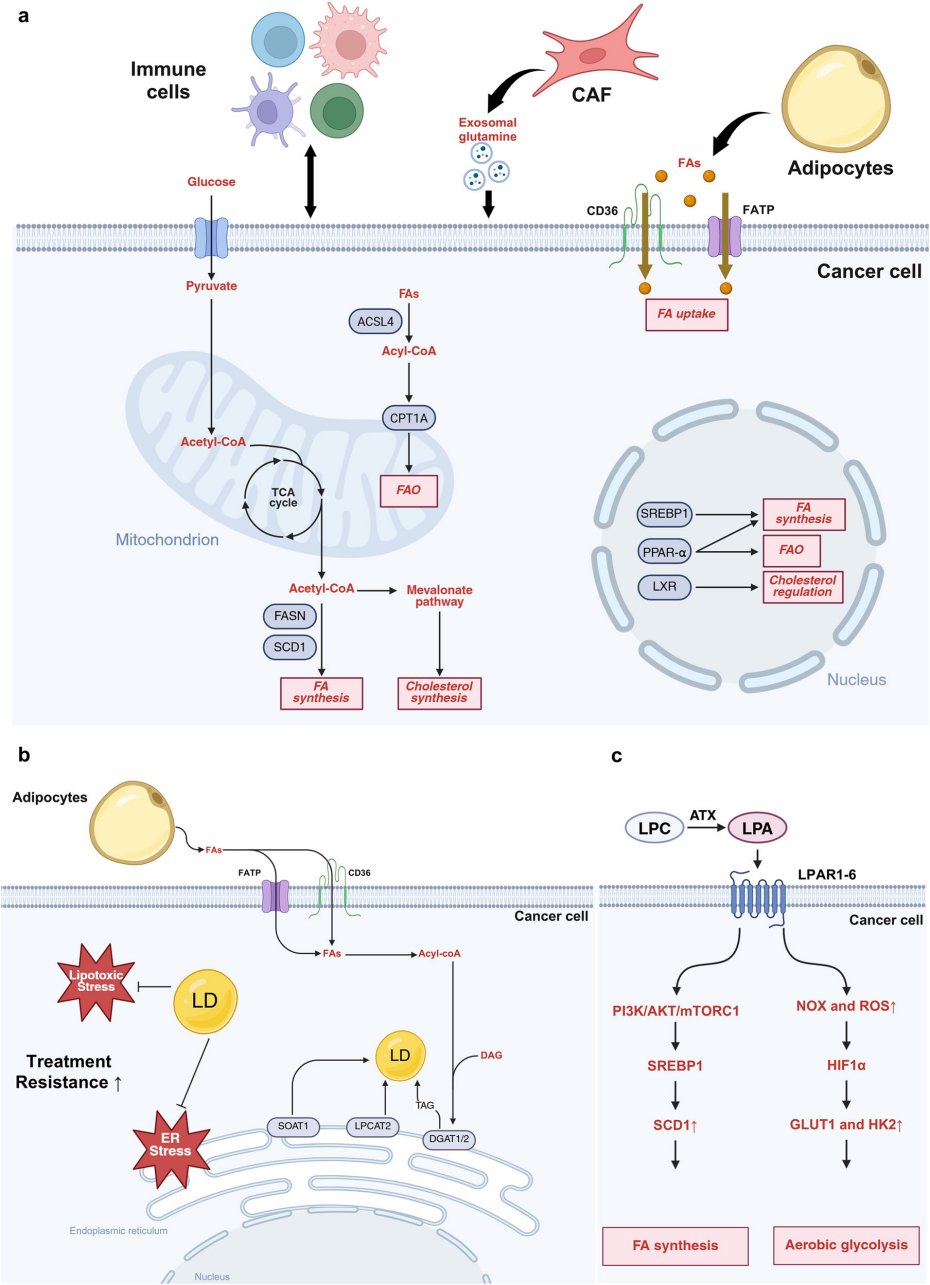
Heterogeneity of lipid metabolism and its functional implications in the TME. **a** Cancer cells display enhanced de novo fatty acid (FA) synthesis and increased uptake of exogenous lipids from the TME to sustain growth and survival. FAs taken up through CD36 and FATP transporters are metabolized to acyl-CoA by ACSL and used either for β-oxidation via CPT1A or for anabolic lipid synthesis. Acetyl-CoA, derived from glucose or lipid sources, integrates glycolysis and mitochondrial metabolism, fueling the TCA cycle, FA synthesis (FASN, SCD1) and the mevalonate–cholesterol biosynthetic pathway. Transcriptional regulators such as SREBP1, PPAR-α and LXR coordinate these pathways to balance lipid synthesis, oxidation and cholesterol homeostasis in cancer cells. **b** FAs provided by adipocytes promote CD36/FATP-dependent uptake, driving acyl-CoA formation and LD biogenesis. Neutral lipids are synthesized via DGAT1/2 and LPCAT2 and stored in LDs, mitigating ER and lipotoxic stress. The accumulation of LDs and cholesterol esters (via SOAT1) through these pathways confers resistance to metabolic and chemotherapeutic stress. These lipid storage programs protect cancer cells from oxidative damage and apoptosis, facilitating therapeutic tolerance. **c** LPA, generated from LPC by ATX, signals through LPA receptors to activate PI3K–AKT–mTORC1 and NOX/ROS–HIF-1α pathways. These cascades promote SREBP1-dependent lipogenesis and upregulate glycolytic enzymes (GLUT1, HK2), establishing a feed-forward loop that enhances FA synthesis and aerobic glycolysis. LPA signaling thereby integrates metabolic reprogramming and proliferative signaling, reinforcing tumor progression and resistance within the heterogeneous TME.

Nonmalignant cells in the TME—including adipocytes, fibroblasts and diverse immune subsets—undergo profound lipid metabolic rewiring that shapes nutrient sharing, inflammation, antigen presentation and immune tolerance (Fig. 3a). These adaptations often promote an immunosuppressive or protumoral state^134^.

Adipocytes located near tumors act as dynamic lipid reservoirs, releasing free fatty acids that fuel tumor growth and metastasis^135^. In breast cancer, mammary adipocytes enhance invasion and migration through the metabolic remodeling of adjacent tumor cells^136^. CAFs similarly fuel cancer metabolism by secreting metabolites—including glutamine-containing exosomes—that support reductive lipid synthesis and enhance colorectal cancer cell migration^137,138^. In metabolically stressed environments, CAFs also stimulate fatty acid catabolism in tumor cells, thereby promoting peritoneal metastasis in colon cancer^139^.

Immune cells in the TME face nutrient competition with highly glycolytic tumor cells and often compensate by relying on FAO for energy generation^140^. Among these, TAMs are strongly influenced by lipid metabolism. Distinct metabolic programs drive polarization toward M1 (antitumor) or M2 (protumor) phenotypes^141^. Enhanced lipid biosynthesis and uptake promote M2-like protumoral activity, whereas lipid-laden macrophages and foam cell-like TAMs emerge as key immunosuppressive populations that transfer lipids to tumor cells, supporting their anabolic and energetic needs^142,143^.

T_reg_ cells also depend on lipid metabolic adaptation to survive within the hostile TME^144^. The CD36-mediated uptake of oxidized lipids promotes T_reg_ persistence and function^145^, whereas excessive lipid accumulation in CD8^+^ T cells impairs effector function and fosters exhaustion^146^. Similarly, tumor-derived lipids disrupt DC function by inducing lipid accumulation and endoplasmic reticulum (ER) stress, leading to impaired antigen presentation^147^. The inhibition of PPAR-α signaling has been shown to restore DC activity and counteract tumor-derived exosomal lipid effects^148^.

Collectively, stromal and immune lipid programs act not as bystanders but as codrivers of tumor progression. Therapeutic strategies that normalize adipocyte- and CAF-derived lipid fluxes while restoring T cell and DC metabolic fitness represent a promising frontier for reversing immunosuppression and improving antitumor efficacy.

### Lipid transfer and storage niches as resistance strategies

Cancer cells frequently evade therapy by importing lipids to fuel β-oxidation and by storing excess fatty acids and cholesterol esters in LDs, which mitigate lipotoxic stress and rewire signaling pathways to promote treatment resistance (Fig. 3b). The accumulation of LDs correlates with, and functionally contributes to, resistance across cancer types through the sequestration of surplus fatty acids, attenuation of ER stress and caspase activation and reduction of oxidative lipotoxicity during therapy^149^.

Adipocyte–tumor metabolic crosstalk further amplifies this resistance. Omental and subcutaneous adipocytes, responding to tumor-derived lipolytic cues, release long-chain fatty acids that are imported by tumor cells via CD36 and fatty acid transport proteins (FATPs/SLC27), fueling growth and therapy resistance^150^. In ovarian cancer, adipocytes induce CD36 expression in tumor cells, accelerating fatty acid uptake and driving aggressive phenotypes; the blockade of this axis suppresses proliferation and resensitizes cells to chemotherapy^151^. Multiple FATP isoforms are similarly upregulated across solid tumors, forming a broad transporter program that enhances exogenous lipid influx and metabolic flexibility under therapeutic stress^152^.

Adipocyte-derived factors also trigger FABP4 induction in ovarian cancer cells upon the colonization of lipid-rich omental niches. FABP4 supports metastatic adaptation and carboplatin resistance, defining an adipocyte–tumor lipid-handling axis that mediates chemoprotection^153^. In colorectal cancer, the LD-associated enzyme LPCAT2 remodels phosphatidylcholine and drives LD biogenesis; elevated LD content confers resistance to 5-fluorouracil and oxaliplatin by dampening unfolded protein response-mediated apoptosis^154^. Similar LPCAT2–LD circuits have been identified in pancreatic tumors, where STAT-dependent signaling promotes LD accumulation and chemoresistance, suggesting a recurring paradigm of storage-mediated survival^155^.

Cancer cells also exploit triacylglycerol and cholesterol esterification pathways to neutralize toxic lipids. DGAT1/2, the terminal enzymes of triacylglycerol synthesis, package fatty acids into LDs; the genetic or pharmacologic inhibition of DGAT reduces LD formation and sensitizes tumor cells to oxidative and glucose stress^156^. In glioblastoma, SOAT1-dependent cholesterol esterification produces cholesteryl ester-laden LDs; targeting SOAT1 suppresses SREBP1-driven lipogenesis, curtails tumor growth and extends survival in xenograft models, implicating sterol storage as both a biomarker and therapeutic target^157^.

Collectively, these findings establish lipid transfer and storage as key adaptive resistance circuits in cancer. Disrupting fatty acid uptake (for example, via CD36 or FATP inhibition) and LD homeostasis (for example, through LPCAT2 or DGAT blockade), when combined with standard chemotherapies, offers a promising strategy to overcome metabolic plasticity and drug resistance.

### LPA signaling in the TME

LPA is a pleiotropic lysophospholipid mediator that signals through G protein-coupled receptors (LPAR1–LPAR6) to regulate cell proliferation, survival, motility, EMT and stromal–immune interactions^158^. In many solid tumors, LPA levels are elevated owing to the enzymatic activity of autotaxin (ATX; ENPP2), which converts lysophosphatidylcholines (LPCs) in the TME into LPA. This creates a self-sustaining extracellular catalytic loop that fuels oncogenic signaling and contributes to therapeutic resistance^159^. Recent evidence highlights the ATX–LPA–LPAR axis as a key metabolic and signaling node integrating lipid rewiring, stemness maintenance and niche crosstalk in diverse tumor types, including CSCs^160^ (Fig. 3c).

ATX-mediated LPA release activates AKT signaling, promoting the proliferation and directional migration in ovarian CSCs^161^. The inhibition of ATX—either genetically or pharmacologically—suppresses PDAC growth and invasion, underscoring the stromal contribution of lysolipids to tumor maintenance^162^. Mechanistically, LPA stimulates PI3K–AKT–mTORC1 signaling, leading to the proteolytic activation of SREBP1 and upregulation of FASN, ATP-citrate lyase (ACLY) and acetyl-CoA carboxylase (ACC)^163^. In parallel, LPA enhances aerobic glycolysis by inducing hexokinase 2 (HK2) and other hypoxia-inducible gene products, partly through a pseudohypoxic state generated by reactive oxygen species and HIF-1α stabilization. The pharmacological inhibition of HK2 reduces tumor burden in ovarian cancer xenografts, confirming LPA’s role in metabolic reprogramming^164^.

LPA also acts as a potent chemoattractant, driving organotropic metastasis. In breast cancer, platelet-derived LPA promotes osteolytic bone metastasis through LPAR1, and small-molecule LPAR1 antagonists effectively suppress metastatic spread in preclinical models^125^. In PDAC, N-WASP-regulated trafficking of LPAR1 determines receptor recycling and surface availability, enabling chemotactic gradient sensing and metastatic competence. Disrupting this trafficking program reduces invasion and in vivo dissemination^165^, establishing LPAR blockade and receptor trafficking interference as viable antimetastatic strategies.

Beyond its tumor-intrinsic roles, ATX-generated LPA profoundly modulates antitumor immunity. In melanoma, tumor-secreted ATX induces chemorepulsion of CD8⁺ T cells via LPAR6, limiting effector infiltration and impairing vaccine-induced tumor regression^166^. Moreover, LPA signaling reshapes T cell metabolism and promotes exhaustion, positioning LPA as a lipid checkpoint that regulates effector function and spatial distribution within tumors^167^.

Collectively, these findings establish the ATX–LPA axis as a central orchestrator of proliferative, invasive and immunomodulatory signaling in the TME. Therapeutically, disrupting LPA production or receptor signaling holds promise for simultaneously targeting tumor growth, metastatic dissemination and immune suppression, thereby enhancing the efficacy of conventional and immune-based cancer therapies.

### Crosstalk between amino acid and lipid metabolism in the TME

Glutaminolysis provides a direct carbon bridge into lipid anabolism by supplying TCA cycle intermediates and sustaining citrate levels, which can be converted into acetyl-CoA. Glutamine-derived acetyl-CoA has been reported to serve as a precursor for malonyl-CoA formation, thereby supporting de novo fatty acid synthesis and membrane biogenesis^168,169^. Notably, this carbon rerouting is not restricted to cancer cells; stromal cells can facilitate this process, as CAF-derived exosomes deliver amino acids and TCA cycle metabolites that are readily utilized by cancer cells to promote glutamine-dependent reductive carboxylation and drive fatty acid synthesis^98^.

Beyond providing carbon substrates, BCAAs and glutamine availability can be translated into lipid metabolic output through nutrient-sensing pathways, most prominently, mTORC1. Lysosome-dependent scavenging of ECM proteins replenishes intracellular amino acid pools to activate anabolic transcriptional programs via mTORC1^90^. Sustained mTORC1 activity subsequently induces SREBP1-driven lipogenesis, upregulating lipogenic enzymes including ACLY, ACC, FASN and SCD1^163^. This illustrates an additional axis of amino acid–lipid crosstalk mediated by mTORC1.

Amino acid and lipid metabolism also intersect in the regulation of redox homeostasis and ferroptosis sensitivity^163^. Cysteine and glutamine-derived GSH modulate ferroptosis, an iron-dependent lipid peroxidation process. The lysosomal degradation of scavenged proteins such as albumin and ECM components provides lipid substrates and influences cellular redox balance, thereby determining the extent to which lipid peroxides accumulate to trigger ferroptosis in cancer cells.

Collectively, the interplay between amino acid and lipid metabolism highlights the vulnerability of single-pathway inhibition, which is prone to compensatory rewiring. Targeting such metabolic crosstalk—for example, the glutamine–lipid anabolic axis or the mTROC1–SREBP1 nutrient-sensing module—may therefore offer improved therapeutic strategies to eradicate tumors that exploit metabolic flexibility within the TME.

## Novel therapeutic strategies

A growing body of evidence indicates that the efficacy of targeting amino acid metabolism is profoundly shaped by the TME, owing to its dense network of interactions among cancer cells, stromal components and immune populations^9^. These intercellular interactions—mediated through cytokine signaling, nutrient competition and exosome trafficking—create a metabolically and immunologically specialized niche that fosters immune evasion and therapeutic resistance.

Within this network, CAFs and MDSCs play pivotal roles in remodeling metabolic fluxes and shaping immune function. CAFs modulate glutamine availability through exosomal transfer, whereas MDSCs mediate arginine depletion, collectively suppressing T cell activation and sustaining tumor proliferation. These crosstalk mechanisms not only reprogram nutrient distribution but also interact with immune checkpoint pathways, reinforcing immunosuppressive signaling within the TME.

TME-driven metabolic plasticity further generates inadequate and heterogeneous nutrient availability through nutrient scavenging mechanisms that bypass canonical transporter-mediated uptake. Cancer cells can catabolize extracellular macromolecules through macropinocytosis to replenish intracellular amino acid pools and sustain anabolism under hypoxia and nutrient scarcity^170,171^. This mechanism contributes to therapeutic resistance by maintaining tumor growth when nutrient availability or specific metabolic pathways are pharmacologically restricted, effectively compensating for targeted metabolic inhibition.

In parallel, metabolic coupling with CAFs provides additional bypass routes. CAFs remodel the ECM to generate physical barriers and secrete factors that promote cancer cell survival by altering redox handling and enabling the use of alternative substrates, thereby conferring resistance to chemotherapy and immunotherapy^172,173^. Spatial immune exclusion, in which malignant tumors and stromal cells restrict physical contact between effector lymphocytes and tumor nests, has also been revealed through single-cell and spatial profiling approaches that identified tumor programs driving T cell exclusion and determining immunotherapy responses^174,175^. These findings demonstrate that therapeutic response depends not only on immune abundance and polarization but also on the spatial segregation of distinct cell populations.

**Fig. 4:**
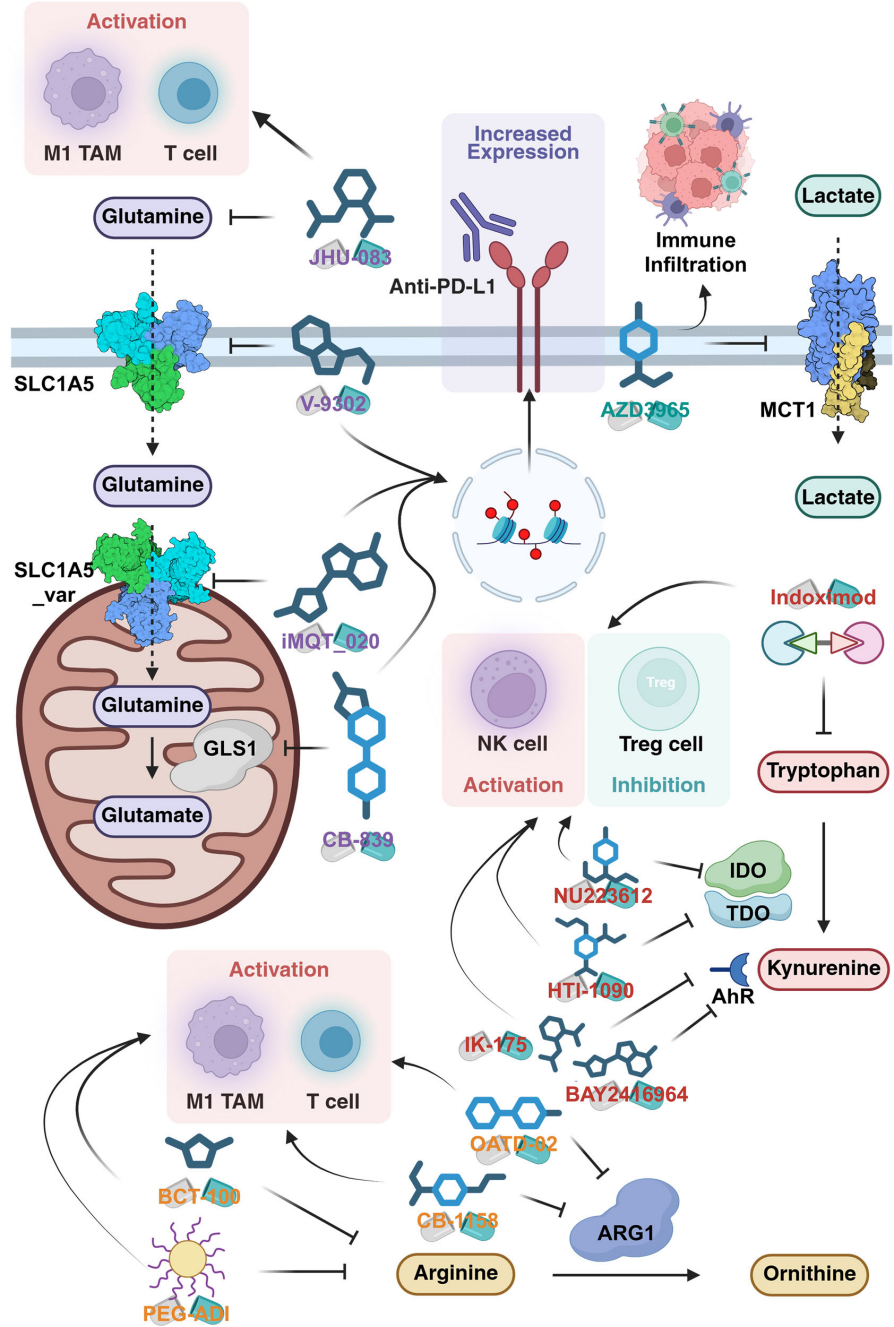
Therapeutic strategies involving amino acids of the TME. Multiple metabolic pathways that sustain tumor growth and immune evasion are being therapeutically exploited. Arginine depletion by ADI-PEG and BCT-100 promotes M1 macrophage polarization, T cell activation and tumor cell apoptosis. The inhibition of tryptophan catabolism with indoximod, NU223612 (a PROTAC for IDO), HTI-1090, BAY2416964 or IK-175 blocks the IDO/TDO–AHR–kynurenine axis, thereby preventing T_reg_ induction and promoting NK and MDSC activation and starvation of tryptophan in cancer cells. Targeting lactate transport with AZD3965 increases immune infiltration by disrupting MCT1-mediated export. Arginase inhibitors (OATD-02 and CB-1158) counteract immunosuppressive arginine depletion by MDSCs and TAMs, restoring effector T cell function. Glutamine antagonists such as JHU-083 suppress tumor metabolism and increase the activity of cytotoxic T cells. V-9302, CB-839 and iMQT_020, which are selective mitochondrial glutamine transporter inhibitors, reduce α-KG availability, altering epigenetic regulation and maintaining PD-L1 expression. These inhibitors, in combination with immune checkpoint inhibitors (anti-PD-L1), have synergistic effects on the killing of cancer cells. Therapeutic strategies to inhibit features of the TME are effective at starving cancer cells and activating the immune system to effectively kill cancer cells.

Consequently, disrupting these reciprocal metabolic exchanges—particularly those that bridge tumor metabolism and immune regulation—has emerged as a cornerstone of multitarget therapeutic strategies. Approaches that simultaneously inhibit metabolic dependencies and restore immune competence are gaining traction as the next frontier in precision oncology (Fig. 4).

### Targeting amino acid metabolism in the TME

Among the diverse metabolic nodes regulating amino acid biology, arginine and glutamine have emerged as key therapeutic targets. Arginine-depleting strategies, including PEGylated ADI (ADI-PEG20) and arginase-based enzymes such as BCT-100, have demonstrated efficacy in arginine-auxotrophic tumors, notably in hepatocellular carcinoma and melanoma^18,72^. Conversely, arginase inhibitors such as CB-1158 restore arginine availability in the TME, promote M1-like macrophage polarization and enhance T cell infiltration, collectively improving antitumor immunity. These dual modalities highlight the complex role of arginine metabolism in supporting both tumor growth and immune regulation.

Glutamine antagonism represents another major therapeutic avenue, with interventions designed to disrupt cellular glutamine uptake (V-9302), mitochondrial transport (iMQT_020), GLS activity (CB-839) or overall glutamine flux (JHU-083)^60,176^. These approaches target the metabolic foundations of cancer—impairing nucleotide biosynthesis, redox homeostasis and mTOR signaling. Notably, glutamine inhibition can also reprogram immune phenotypes, as glutamine-limiting conditions enhance T cell effector function and synergize with immune checkpoint blockade^111^.

However, therapeutic design must carefully account for the differential metabolic requirements of tumor and immune cells. Although glutamine deprivation suppresses tumor proliferation, it can also impair antigen presentation and CD8⁺ T cell expansion if not spatially or temporally controlled^21^. To overcome this challenge, advanced drug delivery systems—including nanoparticle-based vehicles, TME-specific prodrugs and tumor-targeted conjugates—are being developed to achieve compartmentalized metabolic modulation.

Recent studies demonstrate that inhibition of glutaminolysis—either at the level of cellular uptake (V-9302)^177^ or mitochondrial import (iMQT_020)^176^—not only deprives cancer cells of glutamine but also upregulates PD-L1 expression, thereby sensitizing tumors to immune checkpoint inhibitors^178^. This synergy between metabolic inhibitors and immunotherapies exemplifies a promising paradigm in precision oncology, where dual-target interventions can effectively remodel the metabolic–immune interface to achieve durable tumor eradication.

### Targeting lipid and cholesterol metabolism through FASN, SCD1, and PPARα

**Fig. 5:**
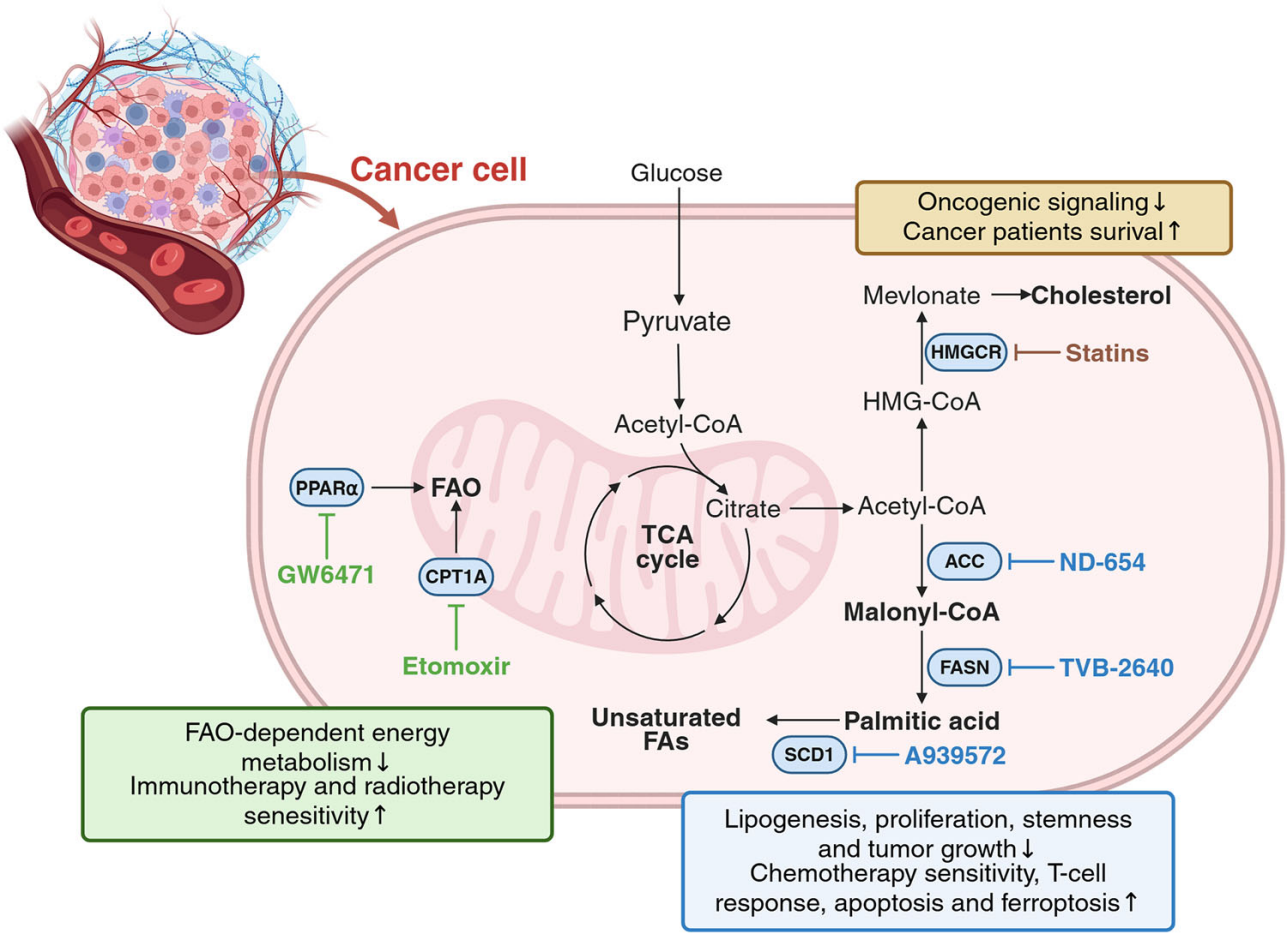
Therapeutic strategies involving lipids of the TME. Pharmacological inhibition of these metabolic choke points suppresses tumor growth and enhances therapeutic responses. The FASN inhibitor TVB-2640 (denifanstat) and ACC inhibitor ND-654 reduce lipogenesis and tumor proliferation, whereas SCD1 inhibition (A939572) decreases monounsaturated fatty acid production, sensitizing tumors to ferroptosis and improving T cell responses. The inhibition of FAO through blockade of CPT1A (etomoxir) or PPARα (GW6471) attenuates mitochondrial FA oxidation and enhances sensitivity to immunotherapy and radiotherapy. Statins, which inhibit HMGCR, suppress tumor growth and have been associated with improved patient survival. Collectively, the inhibition of these lipid metabolic pathways disrupts energy production and biosynthetic capacity while increasing susceptibility to chemotherapy, radiotherapy, ferroptosis and immune checkpoint blockade.

Lipid metabolic reprogramming is a conserved adaptive program across tumor, stromal and immune compartments that supports biosynthesis, membrane remodeling, redox control and therapy resistance. Key enzymatic nodes—including FASN/ACC (de novo synthesis), SCD1 (unsaturation control), CPT1A–FAO/PPARα (mitochondrial import and oxidation) and HMG-CoA reductase (HMGCR)/mevalonate (sterol biogenesis)—have therefore emerged as clinically actionable checkpoints^134^ (Fig. 5).

FASN, the central enzyme for de novo fatty acid synthesis, is overexpressed in numerous malignancies, and its inhibition suppresses proliferation and induces apoptosis^179^. FASN inhibitors such as TVB-2640 (denifanstat) have progressed to clinical trials in high-grade astrocytoma^180^. ACC, the rate-limiting enzyme generating malonyl-CoA for FASN, represents an additional upstream target^168^; its inhibition with ND-654 suppresses lipogenesis and tumor growth in NSCLC and HCC models^181,182^. Moreover, FASN blockade enhances chemotherapy efficacy—for example, the inhibition of FASN sensitizes cervical cancer to cisplatin via xCT-mediated ferroptosis^183^.

SCD1, which converts saturated to monounsaturated fatty acids, provides an antiferroptotic defense and has emerged as another attractive target^184^. SCD1 inhibition suppresses bladder CSC growth and overcomes resistance mechanisms^185^. Importantly, it also enhances antitumor T cell responses and synergizes with anti-PD-1 therapy, underscoring its role in immunometabolic regulation^186^.

The PPARα–FAO axis represents a complementary metabolic vulnerability. PPARα, a nuclear receptor that regulates FAO, supports tumor survival under metabolic stress^187^. PPARα inhibition reverses tumor-derived exosomal lipid-induced DC dysfunction, thereby improving immunotherapy efficacy^148^. Blocking PPARα or its downstream partner CPT1A, the rate-limiting enzyme for mitochondrial fatty acid transport, abrogates FAO-dependent energy metabolism—for instance, in ovarian CSCs^188^—and FAO inhibition with etomoxir sensitizes nasopharyngeal carcinoma to radiation therapy^189,190^.

Finally, targeting cholesterol synthesis, particularly via the mevalonate pathway, represents an additional therapeutic avenue. Statins, which inhibit HMGCR, have demonstrated survival benefits in patients with cancer and modulate membrane composition and oncogenic signaling^133^.

Collectively, these lipid metabolic circuits define a therapeutic architecture in which FASN/ACC control carbon entry into lipogenesis, SCD1 tunes the MUFA–PUFA balance and ferroptosis sensitivity, the PPARα–FAO axis sustains metabolic resilience and the mevalonate pathway governs membrane integrity and oncogenic signaling. Together, they constitute complementary, nonredundant vulnerabilities that can be exploited for combination therapies targeting tumor metabolic plasticity.

### Multitarget approaches

Targeting the metabolic plasticity of noncancerous cells within the TME poses a unique challenge because interventions can exert dual effects on both tumor and immune compartments. To address this complexity, multitarget strategies aim to simultaneously suppress tumor metabolism and restore immune competence.

The tryptophan–kynurenine–AHR axis represents one of the most actively explored dual-target systems. IDO and TDO catalyze the production of kynurenine, which activates the AHR to suppress antitumor immunity. The inhibition of these enzymes not only induces tumor cell death but also enhances cytotoxic T cell activity. For instance, NU223612, a PROTAC-based degrader of IDO, improves survival in brain tumor models by concurrently inhibiting tumor growth and activating immune responses. Similar effects have been reported for HTI-1090 and indoximod, inhibitors of IDO and TDO, respectively. Furthermore, AHR antagonists such as IK-175 and BAY2416964 augment immune cytotoxicity, reinforcing this pathway’s therapeutic potential.

Additional multitarget frameworks involve selective modulation of stromal compartments. Rather than depleting all CAFs, TGF-β pathway inhibition is used to deactivate myCAFs while sparing immune-supportive subsets. Parallel strategies combine metabolic checkpoint blockade, such as CD73 inhibition, with PD-1/PD-L1 immunotherapy, enhancing T cell infiltration and effector function^191^.

Increasingly, therapeutic design is incorporating spatiotemporal targeting of metabolic heterogeneity. HIF-1α inhibitors preferentially act on hypoxic tumor cores, whereas GLUT1 inhibitors such as BAY-876 selectively target oxygenated or perivascular niches^192^. MCT1 inhibitors, exemplified by AZD3965, suppress lactate exchange to simultaneously impair tumor metabolism and enhance immune cell infiltration, serving as archetypal multitarget agents.

Collectively, these strategies illustrate the emerging paradigm of immune–metabolic synergy, wherein the dual inhibition of nutrient pathways and immune checkpoints restores cytotoxic T cell function and overcomes the adaptive resistance encoded within the TME (Fig. 4). This integrative approach represents a cornerstone of next-generation metabolic immunotherapy.

### Limitations and challenges

Despite substantial advances, several limitations continue to hinder the clinical translation of amino acid-targeted therapies^3,9^. First, the metabolic heterogeneity of tumors—spanning interpatient, intratumoral and temporal variations—complicates the prediction of therapeutic responses and limits the reproducibility of outcomes across cancer types. Second, systemic toxicity remains a critical concern because many amino acid-metabolizing enzymes and transporters targeted in cancer are also indispensable for normal tissue homeostasis. Balancing efficacy with safety thus requires precise spatial or compartmentalized delivery systems. Third, the metabolic plasticity of cancer cells enables adaptation under therapeutic stress. Tumors can upregulate compensatory pathways—such as macropinocytosis, autophagy or symbiotic nutrient exchange—to circumvent amino acid restriction and maintain survival. These escape routes undermine the long-term efficacy of single-pathway inhibition.

Furthermore, the absence of robust biomarkers for predicting treatment responsiveness remains a major bottleneck. Ongoing innovations in spatial metabolomics, imaging mass cytometry and single-cell multiomics promise to illuminate the context-specific metabolic dependencies of both tumor and immune cells. Such tools are expected to enable patient stratification and foster the development of precision metabolic interventions, ultimately bringing amino acid-targeted therapies closer to clinical reality.

## Future directions

### Integrating spatial multiomics technologies, single-cell technologies and AI/ML modeling

The metabolic behavior of cancer and immune cells is profoundly heterogeneous across spatial niches and temporal stages within the TME. Emerging technologies—including spatial metabolomics, spatial transcriptomics and single-cell sequencing—are now poised to unravel intercellular communication, nutrient flux and local metabolic states with unprecedented spatial and functional resolution^193^. Despite these advances, spatial metabolomics currently faces challenges of sensitivity, standardization and data integration, particularly for the robust profiling of amino acid metabolism at the single-cell or subcellular level. Integrating these datasets through AI and ML offers a path forward. AI-driven computational modeling can help capture non-linear metabolic networks, infer niche-specific vulnerabilities and predict metabolic bottlenecks under different therapeutic pressures^58^. When combined with single-cell and spatial data, these models can simulate how amino acid and lipid fluxes dynamically reorganize within the TME, incorporating feedback from stromal and immune components. Deeper integration between AI tools and multiomics databases dedicated to metabolic research will be essential for translating these insights into predictive frameworks for precision therapy.

A recent pancancer analysis using single-cell RNA sequencing and spatial metabolomics identified two distinct TME hubs, each enriched for specific immunoreactive cell subtypes that correlated with temporal immunotherapy responses^26^. Similarly, studies of spatial metabolic organization in the liver and intestine have illustrated how local nutrient gradients and compartmentalized metabolism shape cellular specialization, providing a road map for future investigations of metabolic heterogeneity in tumors^193^. Genetic frameworks that amplify heterogeneity may need to be incorporated into future multiomics analysis. Extrachromosomal circular DNA, which can drive variable oncogene copy number^194,195^ and increase intercellular plasticity through uneven mitotic segregation^196^, represents one potential contributor to intratumoral diversity that could intersect with metabolic state switching and therapy resistance^197,198^. Collectively, these technological and computational innovations herald a new era of spatially resolved metabolic medicine, enabling the discovery of context-dependent vulnerabilities and guiding the design of precision metabolic interventions tailored to each tumor ecosystem.

### Network biomarkers and liquid biopsy development

Beyond traditional single-analyte biomarkers, emerging evidence supports the use of multimetabolite panels that integrate amino acid levels, transporter expression, enzyme activity and metabolite ratios as robust diagnostic and prognostic indicators^199^. These multidimensional signatures can reflect the metabolic state of both tumor and immune compartments, capturing the dynamic interplay that defines the TME.

The advent of liquid biopsy platforms—analyzing circulating metabolites, exosomal cargo and metabolism-associated microRNA profiles—offers the opportunity for noninvasive patient stratification, therapeutic response monitoring and early relapse detection. By enabling longitudinal sampling, these technologies can capture temporal shifts in metabolic flux that static tissue biopsies may overlook. However, realizing this potential requires systematic validation across large, diverse cohorts and multiple cancer types. Critical challenges include establishing context-specific reference ranges, controlling for confounding variables such as diet, circadian rhythm and comorbidities and achieving analytical standardization across laboratories.

Integrating metabolic signatures with network-level transcriptomic and immune phenotype data will probably enhance both specificity and biological interpretability, paving the way for precision metabolic diagnostics that mirror the complexity of cancer metabolism in real time.

### Translational challenges in network-level targeting

Although single-pathway interventions—such as arginase inhibitors or glutamine antagonists—have shown therapeutic potential, it is increasingly clear that such monotherapies are insufficient to overcome the metabolic plasticity and intercellular redundancy that characterize the TME. To effectively disrupt the metabolic networks sustaining tumor progression and immune evasion, network-centric strategies that modulate multiple nodes or interconnected pathways simultaneously are required^200^. These may include therapeutic combinations that jointly target key amino acid circuits or block intercellular nutrient trafficking mediated by stromal support, exosome exchange or metabolite recycling.

However, translating these multifaceted approaches into the clinic presents substantial challenges. Foremost among these is the difficulty of achieving selective cytotoxicity—that is, targeting tumor metabolism without impairing the function of immune cells or normal tissues^1^. In addition, most current drug delivery systems struggle to penetrate specialized metabolic niches, such as the hypoxic tumor core or immune-infiltrated periphery, limiting their pharmacodynamic reach. Compounding these limitations is the lack of predictive biomarkers for identifying patients most likely to respond, as well as the metabolic divergence between preclinical models and human tumors, which frequently undermines translational fidelity.

Overcoming these obstacles will require the development of next-generation delivery platforms, including tissue-specific prodrugs, nanocarrier systems and compartment-sensitive diagnostics that enable precise spatiometabolic targeting. Advances in three-dimensional microtumor and organoid models are beginning to bridge the translational gap by revealing microenvironment-specific vulnerabilities relevant for drug discovery^201^. For instance, the MAPK inhibitor doramapimod was shown to suppress tumor growth in three-dimensional systems by targeting CAF-specific DDR1/2–MAPK12–GLI1 signaling, underscoring the therapeutic value of context-dependent targets.

Future research should prioritize the identification and validation of such microenvironment-specific therapeutic targets, integrating systems-level modeling with functional validation to design rational, multitarget interventions that can selectively reprogram the tumor–stroma–immune metabolic network.

### Key questions to be answered

To accelerate the development of amino acid- and lipid-targeted cancer therapies, several pivotal questions must be addressed. (1) How can spatial metabolomics work flows be scaled and standardized to resolve single-cell metabolic fluxes in situ across heterogeneous tumor ecosystems? Addressing this will enable precise mapping of metabolic compartmentalization and reveal the dynamic plasticity of the TME at unprecedented resolution. (2) Which combinatorial metabolite signatures can serve as reliable, noninvasive biomarkers for tumor classification, therapeutic response or minimal residual disease? Progress here requires the integration of multiomics, longitudinal profiling and ML analytics to define context-specific metabolic signatures with clinical utility. (3) What are the most efficient intervention points for selectively disrupting intercellular metabolic cooperation without compromising normal tissue homeostasis? This challenge necessitates not only the identification of pathway-specific vulnerabilities but also the design of targeted delivery systems capable of spatially confined modulation. (4) How can metabolic interventions be optimally combined with immunotherapies, such as checkpoint blockade, to enhance response durability and immune precision? Deciphering these interactions will help coordinate metabolic rewiring with immune activation, maximizing synergistic efficacy.

Collectively, future research addressing these questions will define the next generation of precision metabolic therapies, transforming how cancer metabolism is targeted and integrated into immuno-oncologic frameworks.

## Conclusion

Research on cancer metabolism has evolved far beyond the classical Warburg paradigm, encompassing the metabolic interactions among cancer cells, stromal populations and immune infiltrates that collectively define the TME. Amino acid metabolism lies at the intersection of tumor progression, immune modulation and therapeutic response. This Review highlights how nutrient competition and cooperative exchange within the TME create spatially distinct zones of metabolic advantage and deprivation—governing not only cancer cell survival but also immune effector activity and drug sensitivity.

Metabolic pathways involving glutamine, arginine, tryptophan and BCAAs exemplify the dual roles of amino acids in simultaneously promoting tumor growth and suppressing immunity. Beyond their metabolic functions, these pathways influence chromatin remodeling, epigenetic signaling and transcriptional programs that reinforce malignant and immunosuppressive phenotypes. Such multilayered regulation underscores the limitations of therapies that target metabolism in isolation and instead supports integrative, network-level strategies that account for the heterogeneity and plasticity of the TME.

Although amino acid- and lipid-targeted therapies show promise, their clinical translation remains challenged by intratumoral heterogeneity, systemic toxicity, metabolic redundancy and the absence of predictive biomarkers. Overcoming these obstacles will require spatiotemporally resolved diagnostics, multitarget regimens and precision delivery platforms capable of modulating metabolic circuits within defined tumor niches.

Looking ahead, the convergence of spatial multiomics, single-cell profiling and AI-driven modeling is poised to transform our ability to identify and exploit metabolic vulnerabilities. Yet, key questions remain: how to standardize spatial metabolomics work flows, establish multimetabolite biomarkers, selectively disrupt nutrient sharing and align metabolic modulation with immunotherapy. Addressing these challenges will be pivotal for translating metabolic insights into clinically actionable precision oncology. The next generation of interventions must aim to dismantle not only individual pathways but also intercellular networks that enable tumors to adapt, evade immunity and resist therapy.
